# Identification of feeding apparatus components in a heterotrophic marine flagellate

**DOI:** 10.1242/jcs.264933

**Published:** 2026-09-18

**Authors:** Gillian Clifford, Samuel J. P. Taylor, Midori Ishii, Fernanda Cisneros-Soberanis, Bungo Akiyoshi

**Affiliations:** ^1^ Centre for Cell Biology, School of Biological Sciences, University of Edinburgh, Edinburgh EH9 3BF, UK; ^2^ Discovery Research Platform for Hidden Cell Biology, School of Biological Sciences, University of Edinburgh, Edinburgh EH9 3BF, UK

**Keywords:** Feeding apparatus, Cytostome, Cytopharynx, Flagellar roots, Diplonemids, *Paradiplonema papillatum*

## Abstract

Diplonemids are highly abundant heterotrophic single-celled flagellates that are widespread throughout the oceans of the world. They have a highly complex microtubule-based feeding apparatus (the cytostome–cytopharynx complex) located adjacent to the deep flagellar pocket from which two flagella emerge. The apical papilla is a tongue-shaped structure unique to diplonemids that connects the cytopharynx and the flagellar pocket, the latter of which is formed by reinforcing microtubules (MTR) and two flagellar roots called intermediate and dorsal roots. Here, we report identification of 17 proteins that localize at the feeding apparatus or flagellar apparatus in *Diplonema papillatum*. Using ultrastructure expansion microscopy, we show that Mad2 and its interaction partner MBP65 localize at the MTR, intermediate root and dorsal root. Homologs of proteins that associate with the flagellar apparatus in *Trypanosoma brucei* (PFR2, KMP-11 and BILBO1) localize at the feeding apparatus in *D. papillatum*. We also identify proteins that localize at the apical papilla, MTR, parallel microtubule loop and cytopharynx. By discovering many components of the feeding apparatus in diplonemids, this work forms the foundation to understand the feeding mechanism in these highly abundant marine planktonic organisms.

## Teaser

**Summary:** Diplonemids are highly abundant marine protists with a complex feeding apparatus. This study identifies 17 proteins of the feeding or flagellar apparatuses in the model diplonemid *Diplonema papillatum*.

## INTRODUCTION

**Fig. 1.**
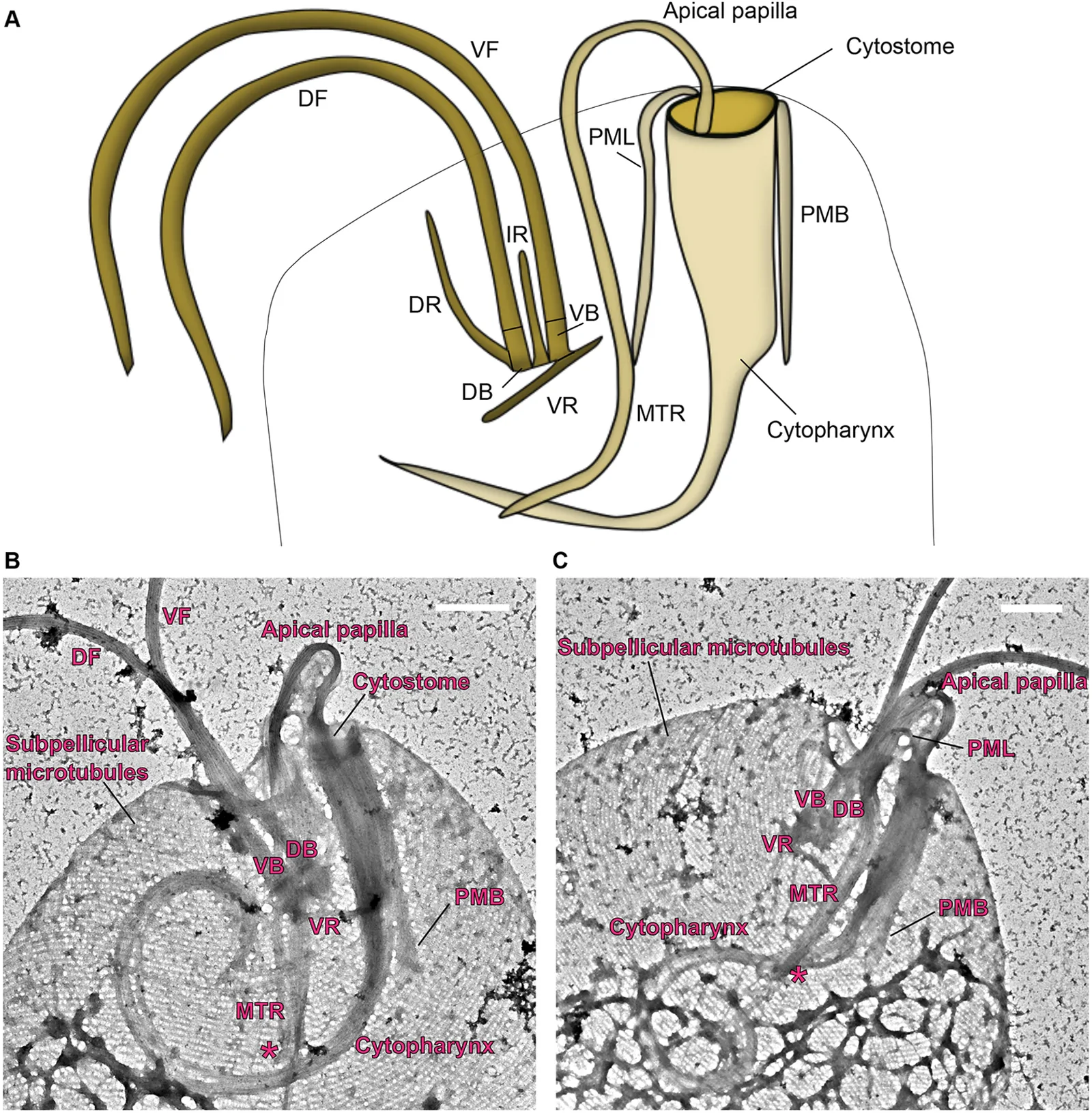
**TEM analysis of detergent-extracted *D. papillatum.*** (A) Cartoon of the anterior part of a *D. papillatum* cell. The thin black line represents the cell body. The flagellar pocket is not depicted. (B,C) *D. papillatum* imaging by TEM. The feeding apparatus locates adjacent to the flagellar apparatus. Cells settled onto a grid were treated with detergent and fixed with glutaraldehyde, followed by a positive staining with uranyl acetate. A connection is observed between the tip of MTR and cytopharynx (marked by asterisks). Note that dorsal root and intermediate root are not readily discernible due to overlap with other structures such as basal bodies and sub-pellicular microtubules. Images representative of two independent repeats. VF, ventral flagellum; DF, dorsal flagellum; VB, ventral basal body; DB, dorsal basal body; VR, ventral root; MTR, reinforcing microtubules; PML, parallel microtubule loop; PMB, peripheral microtubule band. Scale bars: 1 μm.

Obtaining nutrients is a fundamental, universal and continuous activity for all living organisms (Cavalier-Smith, 2013; Leander, 2020). Diplonemids are a group of heterotrophic flagellated eukaryotes that are divergent from traditional model eukaryotes (Burki et al., 2020). They are highly abundant, diverse and widespread throughout the oceans of the world with an inescapable ecological significance (de Vargas et al., 2015; Gawryluk et al., 2016; Flegontova et al., 2016, 2020; Schoenle et al., 2021; Tashyreva et al., 2022; Pilátová et al., 2023). Diplonemids have a highly complex microtubule-based feeding apparatus, characterized by a cytostome (cell mouth) located at the anterior end of the cell, leading into a tubular cytopharynx (gullet) (Montegut-Felkner and Triemer, 1996; Tashyreva et al., 2018, 2023; Prokopchuk et al., 2019) (Fig. 1A). The cytostome–cytopharynx complex locates adjacent to the deep flagellar pocket from which two flagella emerge from basal bodies lying parallel (Porter, 1973). The apical papilla, a tongue-shaped structure unique to diplonemids, connects the cytopharynx and the flagellar pocket. It continues as a structure called reinforcing microtubules (MTR), which contributes to the formation of the flagellar pocket. The MTR originates underneath the flagellar pocket and also creates the flagellar pocket extension (Montegut-Felkner and Triemer, 1994).

In many protists, flagellar roots that associate with basal bodies play various important roles, such as regulation of cell shape, motility and feeding (Moestrup, 2000). Diplonemids have three asymmetrically arranged flagellar roots – a dorsal root (DR), an intermediate root (IR) and a ventral root (VR) (Montegut-Felkner and Triemer, 1994). The DR originates from the dorsal basal body, the IR originates between the dorsal and ventral basal bodies, and the VR is laterally associated with the ventral basal body. The flagellar pocket is reinforced by two microtubule roots (DR and IR) and the MTR (which is not a flagellar root in diplonemids) (Montegut-Felkner and Triemer, 1994; Simpson, 1997). Little is known about the molecular mechanism of the formation or regulation of flagellar roots.

Diplonemids are evolutionarily closely related to kinetoplastids, which include important pathogens such as *Trypanosoma brucei*, which causes African trypanosomiasis, and *Trypanosoma cruzi* which causes Chagas disease (Cavalier-Smith, 2016; Field et al., 2017). A feeding apparatus is present in free-living kinetoplastids (Brugerolle et al., 1979; Tikhonenkov et al., 2021) and some parasitic trypanosomatids, such as *T. cruzi* (Alcantara et al., 2014; Chasen et al., 2019, 2020), whereas it has been lost in many parasitic trypanosomatids including *T. brucei* and *Leishmania* spp. (Halliday et al., 2021; da Cunha-E-Silva et al., 2025). Despite the widespread presence of the cytostome–cytopharynx complex in flagellates and ciliates, relatively little is known about its protein composition or mechanistic understanding (Guerrier et al., 2017; Etheridge, 2022). In diplonemids, basically nothing is known about the composition of the feeding apparatus.

As part of our ongoing effort to dissect mitotic mechanisms in diplonemids (Akiyoshi et al., 2025), we have examined the localization of various proteins by endogenous YFP tagging in genetically tractable *Diplonema papillatum* (also known as *Paradiplonema papillatum* or *Isonema papillatum*) (Faktorová et al., 2020, 2026; Tashyreva et al., 2022; Valach et al., 2023; Hammond et al., 2025). Here, we report the identification of 17 proteins that localize to the feeding apparatus and/or flagellar apparatus.

## RESULTS

### TEM analysis of detergent-treated cells

We first visualized the feeding apparatus in detergent-extracted *D. papillatum* cells. Cells attached to an electron microscopy (EM) grid were treated with detergent and imaged by transmission EM (TEM) (Fig. 1B,C). In these images, cytostome, cytopharynx, apical papilla, VR, MTR, parallel microtubule loop (PML), two flagella and basal bodies were visible. These results confirm that the feeding apparatus of *D. papillatum* is highly similar to that of *Diplonema ambulator* (Montegut-Felkner and Triemer, 1996).

### Mad2 and MBP65 localize at flagellar roots and MTR

**Fig. 2.**
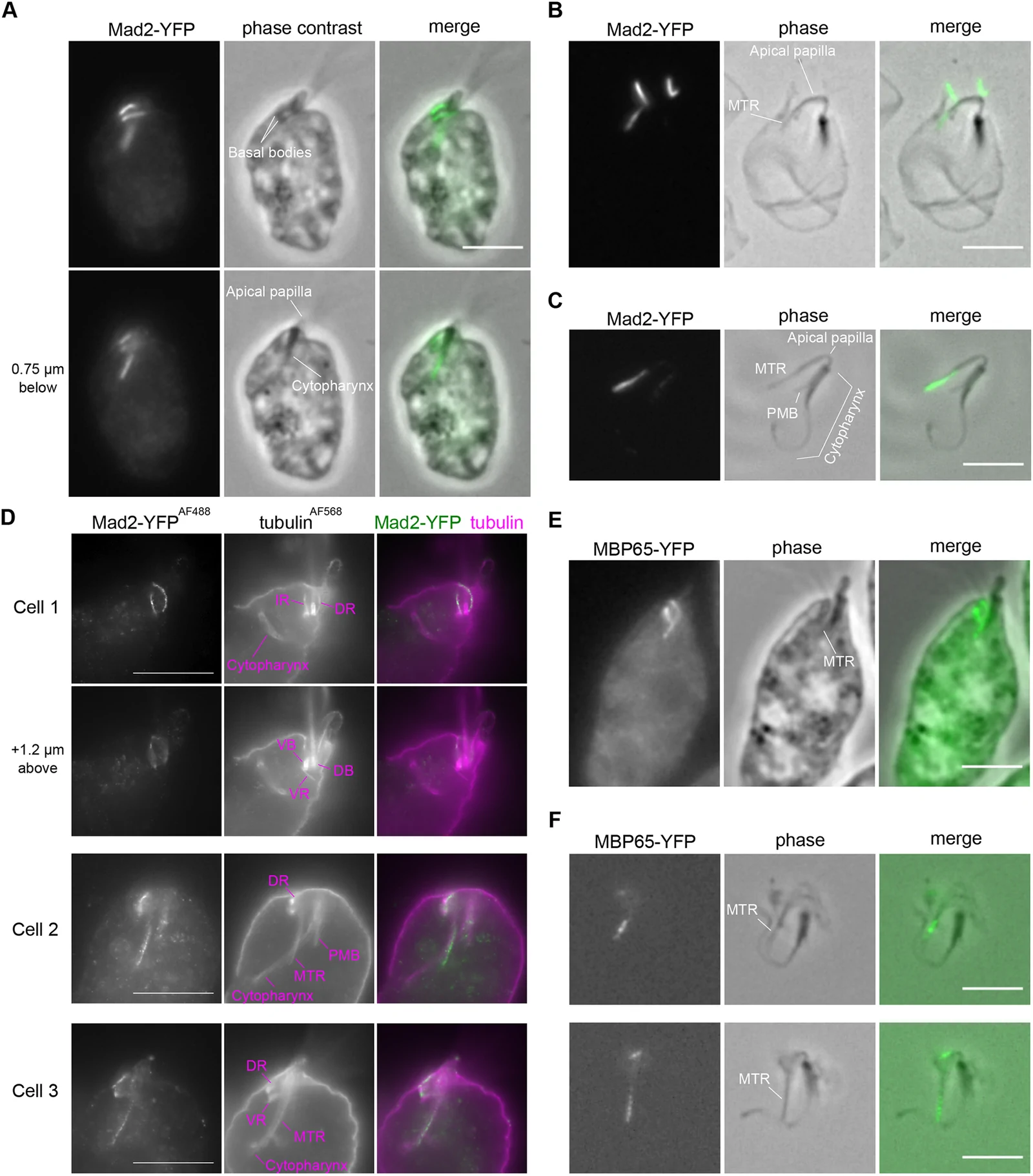
**Mad2 and MBP65 localize at the intermediate root, dorsal root and MTR.** *D. papillatum* imaging by fluorescence microscopy (cell lines, DP6 expressing Mad2–YFP and DP85 expressing MBP65–YFP). (A) Mad2–YFP localizes near basal bodies and the cytopharynx, as previously reported (Akiyoshi et al., 2025). Cells were fixed with formaldehyde and native YFP signal was observed by fluorescence microscopy. Two different *z*-sections are shown. (B,C) Mad2 localizes at a part of the MTR. Cells were treated with detergent, followed by fixation with formaldehyde. (D) U-ExM of cells expressing Mad2–YFP showing its localization at the IR, DR and MTR. Immunostaining was performed using anti-GFP antibody (detected with Alexa Fluor 488; AF488) and anti-tubulin antibody (Alexa Fluor 568: AF568). Two different *z*-sections are shown for cell 1 to highlight the three flagellar roots. The ventral basal body (VB) and dorsal basal body (DB) were determined by the presence or absence of the VR, respectively. Note that the DR originates from the DB. Scale bars in the final view size: 20 μm. (E,F) MBP65 has a similar localization pattern as Mad2. Cells expressing MBP65–YFP without (E) or with (F) detergent treatment were imaged. Images representative of at least two independent repeats. IR, intermediate root; DR: dorsal root; VR: ventral root; DB: dorsal basal body; VB: ventral basal body; MTR: reinforcing microtubules. Scale bars: 5 μm, unless otherwise noted.

In traditional model eukaryotes, such as yeast and human, Mad2 localizes at kinetochores and is involved in the spindle checkpoint control mechanism (Musacchio, 2015). However, in *T. brucei*, Mad2 and its interaction partner MBP65 localize at the microtubule quartet (Akiyoshi and Gull, 2013; Akiyoshi, 2020 preprint; Billington et al., 2023), which is thought to be the remnant of the DR (Lacomble et al., 2009; Yubuki et al., 2016). In *D. papillatum*, the Mad2 homolog localizes near basal bodies and the feeding apparatus (Fig. 2A) (Akiyoshi et al., 2025). To examine its precise localization, we treated cells in a tube with detergent to lyse the cells before imaging (Fig. 2B). When the feeding apparatus was detached from the cell body, Mad2–YFP signal was found on a region of the MTR (Fig. 2C). We next performed ultrastructure expansion microscopy (U-ExM) (Chen et al., 2015; Gambarotto et al., 2019). Immunostaining with an anti-tubulin antibody allowed us to visualize flagellar roots (Fig. 2D). This analysis revealed Mad2–YFP signals on the IR and DR, but not VR. Additional signal was found on a region of the MTR, which seemingly corresponds to the flagellar pocket extension area rather than the flagellar pocket area (Fig. 2D).

We next examined the localization of the MBP65 homolog and found a similar localization pattern (Fig. 2E,F). These results show that Mad2 and MBP65 localize at a subset of flagellar roots in both *D. papillatum* and *T. brucei*. In addition, they localize at the MTR in *D. papillatum*.

### POLO1 localizes at the dorsal root, whereas POLO2 localizes near basal bodies

**Fig. 3.**
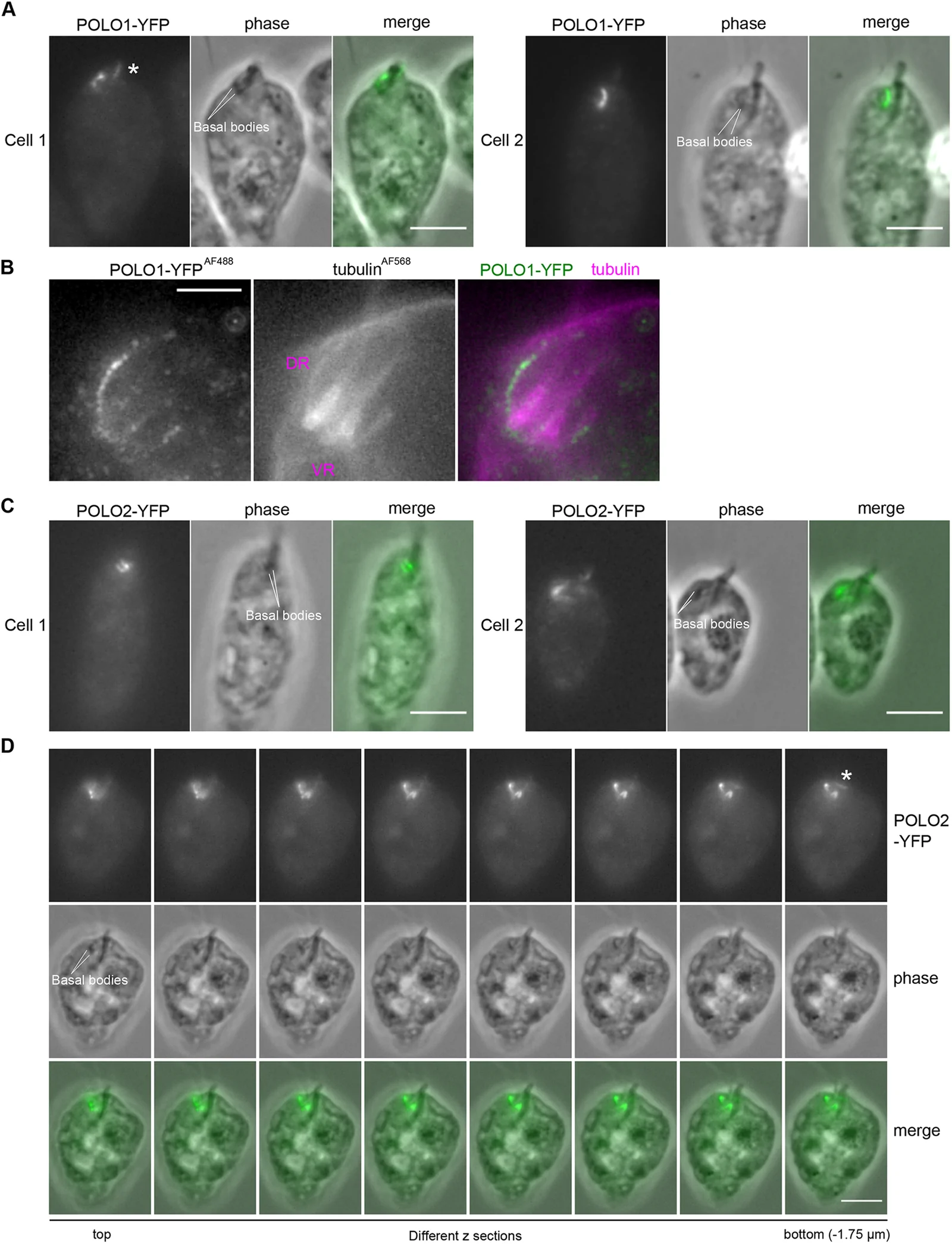
**POLO1 localizes at the dorsal root, while POLO2 localizes at and near basal bodies.** *D. papillatum* imaging by fluorescence microscopy (cell lines, DP10 expressing POLO1–YFP and DP11 expressing POLO2–YFP). (A) POLO1–YFP signal is found near one of the two basal bodies and at an unknown location (asterisk). (B) U-ExM shows that POLO1 localizes at the DR. (C) Two examples of cells expressing POLO2-YFP showing signals near both basal bodies, which may correspond to the DR and IR. (D) *Z*-sections show a complex localization pattern of POLO2 at and near basal bodies, as well as at an unknown location (asterisk). Images representative of at least two independent repeats. Scale bars in the final view size: 5 μm.

Polo-like kinases (PLKs) play diverse functions in eukaryotes (Zitouni et al., 2014). In *T. brucei*, TbPLK is important for the regulation of basal body biogenesis and cytoskeleton (Lozano-Núñez et al., 2013). *D. papillatum* has two proteins that are homologous to polo-like kinases (Benz et al., 2024), named POLO1 (DIPPA_20058) and POLO2 (DIPPA_32534) herein. POLO1–YFP showed signals near one of the two basal bodies and at an unknown location (Fig. 3A). U-ExM revealed that the protein is primarily on the DR, not on the IR (Fig. 3B). POLO2 showed complex localization patterns, including at and around basal bodies (Fig. 3C,D). It is noteworthy that POLO1 and POLO2 signals were found only in a subset of an asynchronously growing cell population. Owing to the lack of cell cycle markers, we were unable to infer cell cycle stages of individual cells. Although we cannot exclude a possibility of inefficient integration of the YFP-tagging constructs at the correct genomic loci, it is plausible that these proteins are cell cycle regulated. Further work is needed to determine the precise location of POLO2 as well as to reveal the functions and cell cycle regulations of the PLKs in diplonemids.

### KMP11A and PFR2

**Fig. 4.**
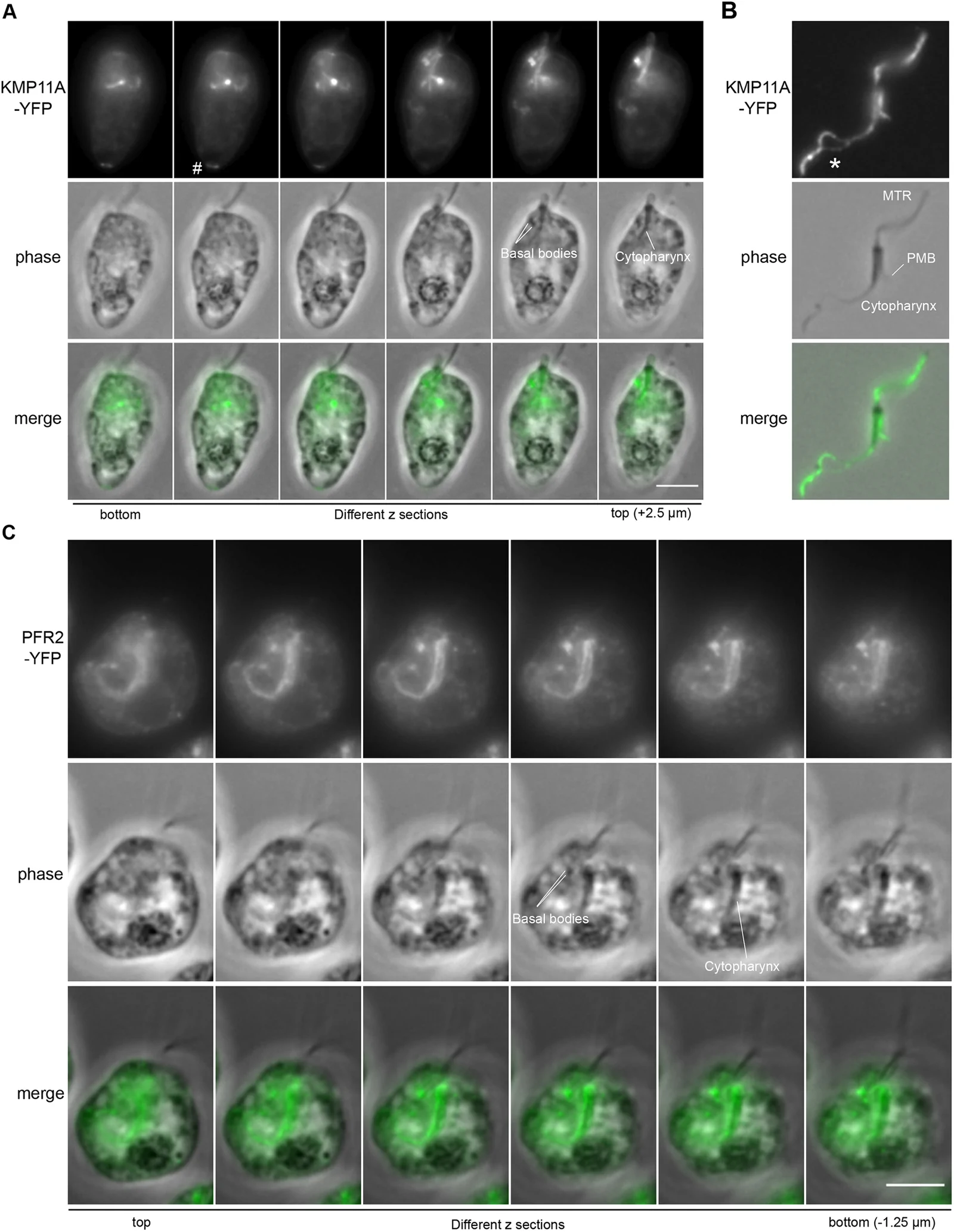
**KMP11A and PFR2 localize at basal bodies and around the feeding apparatus.** *D. papillatum* imaging by fluorescence microscopy (cell lines, DP127 expressing KMP11A–YFP and DP79 expressing PFR2–YFP). (A,B) Cells expressing KMP11A–YFP without (A) or with (B) detergent treatment were imaged. KMP11A has signals at basal bodies, cytopharynx, MTR, and the posterior end (hash). A loop structure is visible in the cytopharynx of detergent treated cells (asterisk). (C) Cells expressing PFR2–YFP shows a similar, but less defined, signal as KMP11A. Additional images are shown in Fig. S1A,B. Images representative of two independent repeats. Scale bars: 5 μm.

Kinetoplastid membrane protein-11 (KMP-11) is an ∼10 kDa protein that is highly conserved among kinetoplastids (Tolson et al., 1994). In *T. brucei*, it localizes at basal bodies and the flagellum (Li and Wang, 2008). Although it was initially thought that KMP-11 is exclusive to kinetoplastids, its homologs are present in diplonemids (Valach et al., 2023). Four KMP-11 homologs are present in the *D. papillatum* genome, termed KMP11A (DIPPA_12089), KMP11B (DIPPA_12088), KMP11C (DIPPA_12130) and KMP11D (DIPPA_12062) herein. KMP11A has 87% sequence identity to the *T. brucei* KMP-11 protein, showing an extremely high level of similarity. KMP11A–YFP signal was found at basal bodies, a region of the flagella, the apical papilla and the MTR, as well as widely at the feeding apparatus including the peripheral microtubule band (PMB) (Fig. 4A,B). It is noteworthy that a ring-like structure was observed in one region of the cytopharynx in detergent-treated cells (Fig. 4B). In addition, KMP11A signals were found at the posterior end of the cell (Fig. 4A) and at unknown cytoplasmic locations (Fig. S1A).

The paraflagellar rod is a paracrystalline filamentous network that runs alongside the axoneme. In *T. brucei,* paraflagellar rod protein 1 and 2 (PFR1 and PFR2) are major components of the paraflagellar rod (Portman and Gull, 2010). Interestingly, although *D. papillatum* lacks a paraflagellar rod (Porter, 1973), their homologs are present in the genome. We found that *D. papillatum* PFR2 (DIPPA_04871, which shares 77% and 69% identity to *T. brucei* PFR2 and PFR1, respectively) has a similar localization pattern to that of KMP11A, although the signal looked less defined (Fig. 4C).

### BILBO1 homologs localize at cytoskeletal structures

**Fig. 5.**
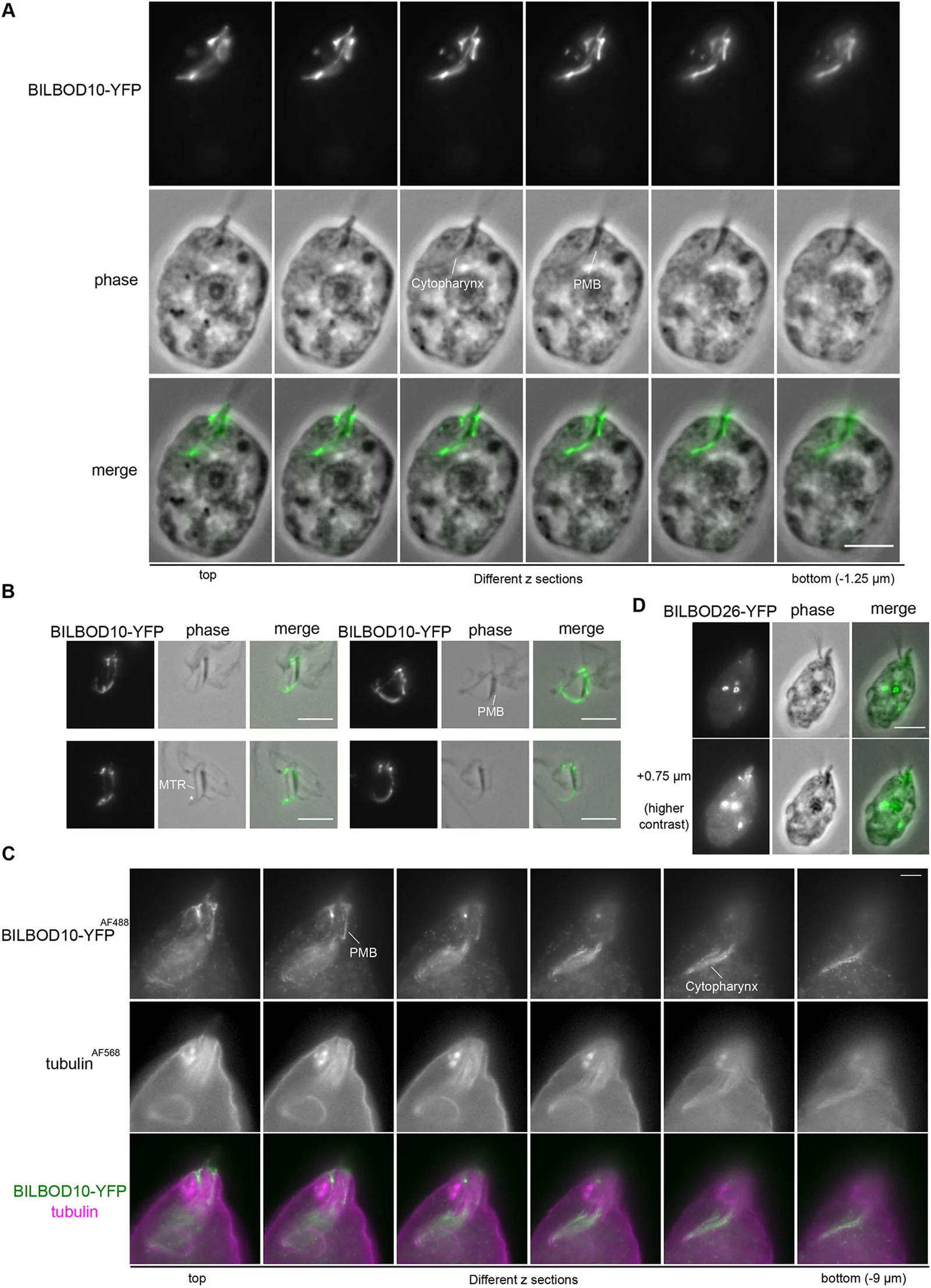
**Two BILBO1 homologs localize near the feeding apparatus*.*** *D. papillatum* imaging by fluorescence microscopy (cell lines, DP100 expressing BILBOD10–YFP and DP99 expressing BILBOD26–YFP). (A,B) BILBOD10 has a complex localization around the feeding apparatus. Cells expressing BILBOD10–YFP without (A) or with (B) detergent treatment were imaged. (C) U-ExM confirms the localization of BILBOD10 at the feeding apparatus area. (D) BILBOD26–YFP shows circle signals in the cytoplasm as well as a weaker signal around the feeding apparatus. Additional images for BILBOD10 are shown in Fig. S1C,D. Images representative of two independent repeats. Scale bars in the final view size: 5 μm.

BILBO1 is a component of the flagellar pocket collar in *T. brucei*, and has polymer-forming properties that allow it to self-assemble into extended filaments (Bonhivers et al., 2008; Florimond et al., 2015; Zelená et al., 2025). *T. brucei* has at least four proteins that have similarities to BILBO1, all of which contain a ubiquitin-like N-terminal domain (NTD) (Vidilaseris et al., 2020). Although BILBO1-like proteins were thought to be kinetoplastid-specific, a large number of homologs are present in diplonemids. *D. papillatum* has 76 proteins that have similarities to the BILBO1 NTD as well as at least ten proteins that have similarities in other regions of BILBO1 (see Materials and Methods). We suggest calling them BILBOD1–BILBOD86 (File S1 at https://doi.org/10.6084/m9.figshare.32409306). We examined the localization of two proteins, BILBOD10 and BILBOD26. BILBOD10–YFP had a complex localization pattern, including at the PMB, a part of cytopharynx, and the connection between cytopharynx and MTR (Fig. 5A–C). In contrast, BILBOD26–YFP showed circular signals of unknown locations as well as signals around the feeding apparatus (Fig. 5D). It will be interesting to examine the localization of other BILBOD proteins.

### PTP2 localizes at apical papilla and PML

**Fig. 6.**
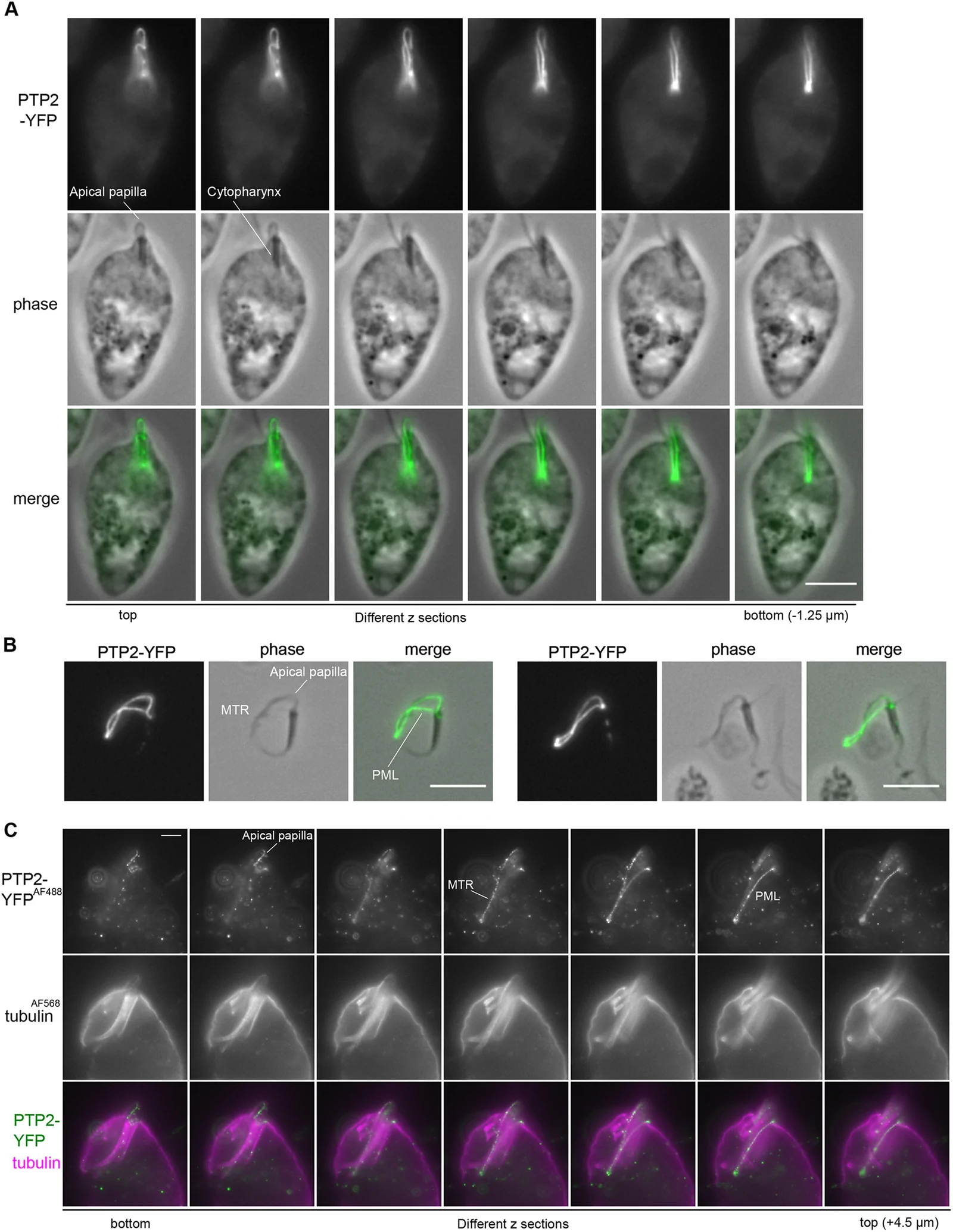
**PTP2 localizes at apical papilla and PML.** *D. papillatum* imaging by fluorescence microscopy (cell line, DP98 expressing PTP2–YFP). (A,B) PTP2 localizes at apical papilla (including MTR) and PML. Cells expressing PTP2-YFP without (A) or with (B) detergent treatment were imaged. (C) U-ExM showing PTP2–YFP. Additional images are shown in Fig. S2. Images representative of two independent repeats. Scale bars in the final view size, 5 μm.

Protein tyrosine phosphatases (PTPs) are a diverse family of enzymes that remove phosphate groups from tyrosine residues on proteins, acting as crucial regulators of cell signalling, proliferation and differentiation (Tautz et al., 2013). There are two PTPs in *D. papillatum*, termed PTP1 (DIPPA_30009) and PTP2 (DIPPA_18426) herein. We found that PTP2 localizes at the apical papilla, MTR and PML (Fig. 6A–C). These images reveal that the PML contacts the side of the MTR as well as its tip.

### Other components that localize at feeding apparatus

**Fig. 7.**
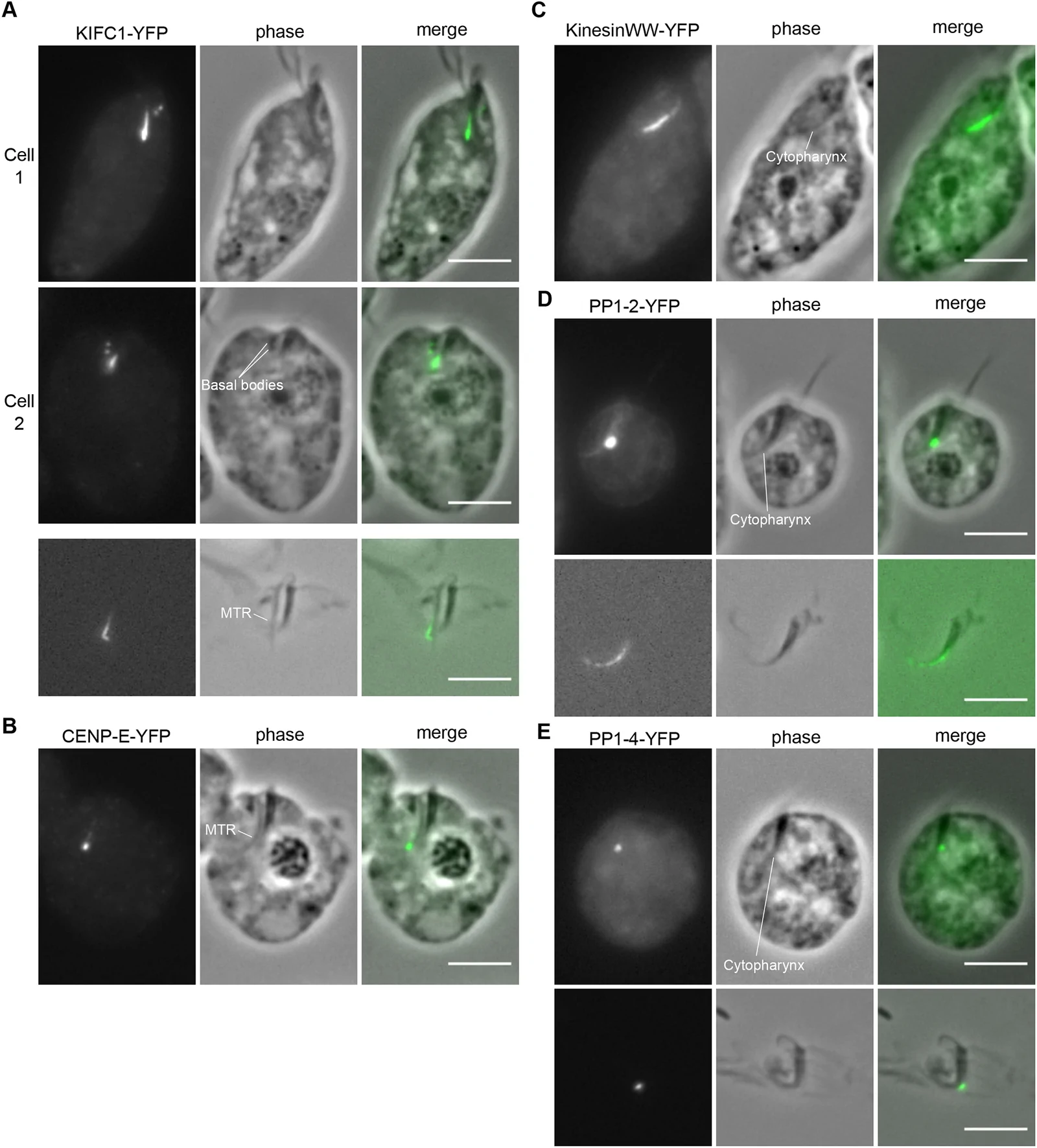
**Identification of five proteins that localize to the feeding or flagellar apparatus.** *D. papillatum* imaging by fluorescence microscopy (cell lines, DP97 expressing KIFC1–YFP, DP31 expressing CENP-E–YFP, DP82 expressing KinesinWW-YFP, DP24 expressing PP1-2–YFP and DP123 expressing PP1-4–YFP). (A) KIFC1–YFP localizes at basal bodies and the MTR. Top and middle, non-treated cells; bottom, detergent-treated cell. (B) CENP-E–YFP localizes near the tip of the MTR. (C) KinesinWW–YFP localizes at the bottom part of the cytopharynx. (D) PP1-2–YFP localizes at cytopharynx as well as near the region of cytopharynx where PMB terminates. Top, non-treated cell; bottom, detergent-treated cell. Note that only weak cytopharynx signal is visible in detergent-treated cells. (E) PP1-4–YFP shows signal near the region of cytopharynx where PMB terminates. Top, non-treated cell; bottom, detergent-treated cell. Images representative of at least two independent repeats. Scale bars: 5 μm.

We identified eight additional components that localize at or near the feeding apparatus. KIFC1 (DIPPA_32533), a C-terminal kinesin motor protein, localizes at (or near) the MTR as well as basal bodies (Fig. 7A), whereas a putative CENP-E protein (Benz et al., 2024) localizes near the tip of the MTR or PML (Fig. 7B). DIPPA_09231 is a highly degenerate kinesin-like protein that localizes at the bottom of the cytopharynx (Fig. 7C), named KinesinWW herein because it has a WW domain, a protein–protein interaction domain that binds proline-rich motifs (Kay et al., 2000). KinesinWW lacks key residues required for a functional kinesin motor (e.g. the P-loop, switch I and switch II), suggesting that it is not a functional motor. Although it is highly conserved in diplonemids, putative orthologs of KinesinWW that have a WW domain are found only in prokinetoplastids (File S2 at https://doi.org/10.6084/m9.figshare.32409306).

*D. papillatum* has five homologs of protein phosphatase 1, termed PP1-1 (DIPPA_07735), PP1-2 (DIPPA_07733), PP1-3 (DIPPA_25219), PP1-4 (DIPPA_29493) and PP1-5 (DIPPA_21477) herein. PP1-2 localizes at the region of cytopharynx approximately where the PMB terminates as well as the bottom part of cytopharynx (Fig. 7D). By contrast, PP1-4 localizes only at the region of cytopharynx where the PMB terminates (Fig. 7E).

**Fig. 8.**
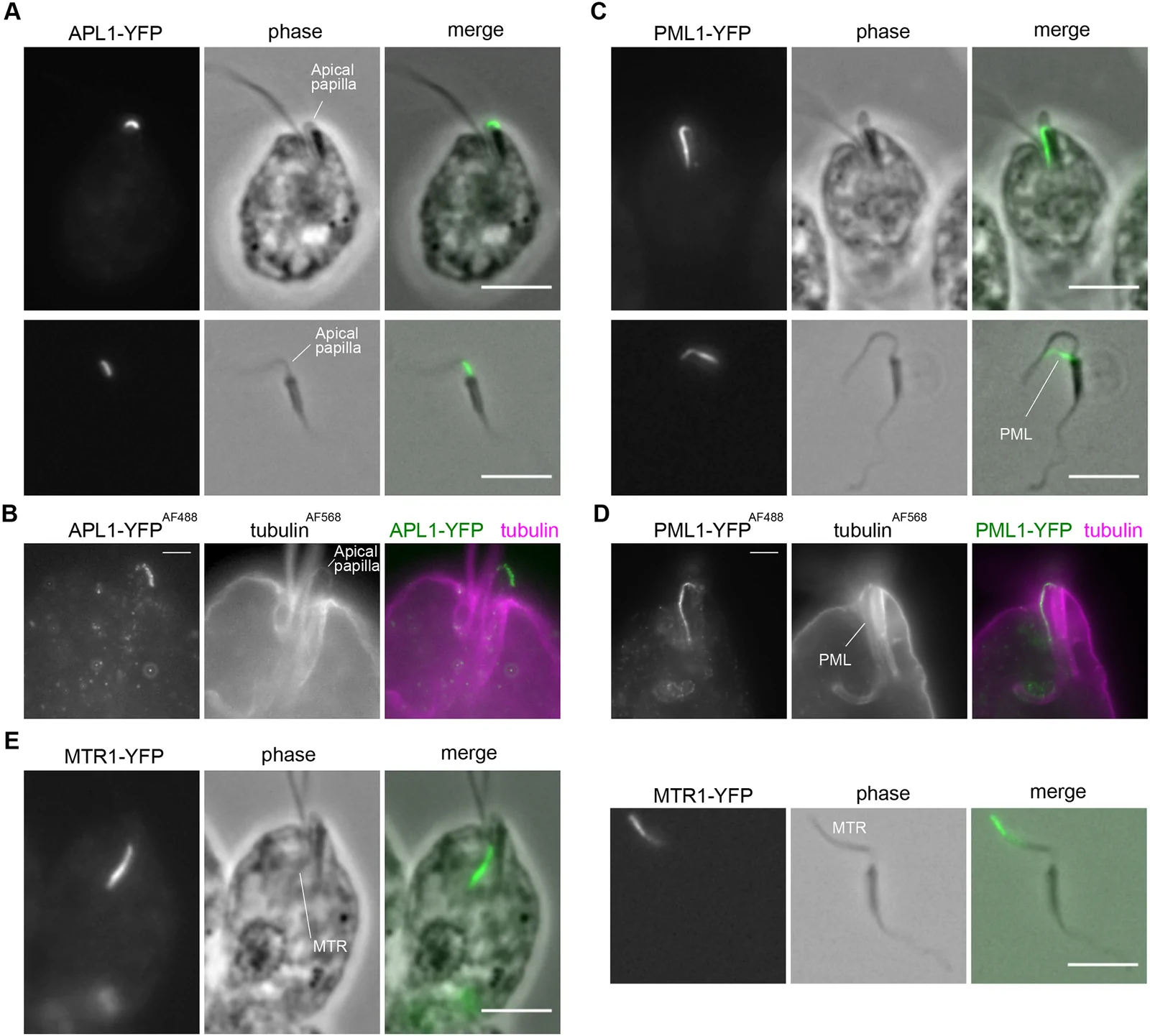
**Proteins that localize at the apical papilla, PML or MTR.** *D. papillatum* imaging by fluorescence microscopy (cell lines, DP61 expressing APL1–YFP, DP119 expressing PML1–YFP, and DP69 expressing MTR1–YFP). (A) APL1–YFP specifically localizes at the beginning of the apical papilla. Top, non-treated cell; bottom, detergent-treated cell. (B) U-ExM showing APL1–YFP. (C) PML1-YFP has signal at the PML. Top, non-treated cell; bottom, detergent-treated cell. (D) U-ExM showing PML1–YFP. (E) MTR1-YFP localizes at the MTR. Left, non-treated cell; right, detergent-treated cell. Images representative of two independent repeats. Scale bars in the final view size: 5 μm.

Finally, by tagging proteins that are conserved among diplonemids but have no annotation, we identified three components of the feeding apparatus. The protein encoded by DIPPA_20809 specifically localizes at the apical papilla (Fig. 8A,B). We have named the protein APL1. Its homologs are present in free-living kinetoplastids and trypanosomatids that have a cytostome-cytopharynx complex (*T. cruzi* and *Crithidia*) (File S3 at https://doi.org/10.6084/m9.figshare.32409306), but not in *T. brucei* or *Leishmania*. The protein encoded by DIPPA_22779 localizes at the PML (Fig. 8C,D), so we have named it PML1. Besides diplonemids, its putative homologs are found only in prokinetoplastids (File S4 at https://doi.org/10.6084/m9.figshare.32409306). PML1 has a caspase-like domain in the N-terminal part of the protein. The protein encoded by DIPPA_13090 localizes at the MTR region (Fig. 8E). We therefore have named it MTR1. Kinetoplastids have MTR1 homologs (File S5 at https://doi.org/10.6084/m9.figshare.32409306), and the *T. brucei* homolog (Tb927.10.630) localizes at the hook complex (Billington et al., 2023).

## DISCUSSION

Determining the composition of macromolecular structures is a key step towards their mechanistic understanding. Previous EM studies have shown that the feeding apparatus is an intricate microtubule-based structure that is conserved among diverse diplonemids (Porter, 1973; Montegut-Felkner and Triemer, 1996; Tashyreva et al., 2018, 2025; Prokopchuk et al., 2019). Experimental tractability of *D. papillatum* makes it an attractive model to identify molecular components of the feeding apparatus in these highly abundant marine flagellates. Furthermore, some components are conserved in disease-causing kinetoplastid parasites, such as *T. cruzi* whose cytostome–cytopharynx complex could be a drug target. Immunoprecipitation of YFP-tagged proteins reported in this work, followed by mass spectrometry has the potential to identify many additional components of the feeding apparatus in *D. papillatum* and beyond.

It is also important to dissect the mechanism that ensures accurate inheritance of the feeding apparatus. Each cell has only one cytostome–cytopharynx complex, implying that it needs to be duplicated and separated properly every time the cell divides. Examining the localization of proteins reported in this study throughout the cell cycle could provide hints into the underlying molecular mechanism.

## MATERIALS AND METHODS

### Cells, plasmids and primers

Plasmids and primer sequences used in this study are provided in Table S1. C-terminal YFP-tagging constructs were made as previously described using pBA3294 (Akiyoshi et al., 2025). Briefly, two ∼2 kb homology arms amplified from genomic DNA by PCR using KOD one polymerase (Merck) were inserted into pBA3294 cut with *Pac*I and *Asc*I with a unique site in between the two PCR fragments (typically *Not*I; if *Not*I was not unique, *Sbf*I or *Spe*I were used as indicated). Plasmids were validated by Oxford Nanopore whole-plasmid sequencing (Plasmidsaurus). As previously observed in our attempts to tag other genes (Akiyoshi et al., 2025), there were occasional mismatches between nanopore sequencing results and expected plasmid sequences, especially in repetitive sequences in 3′ untranslated regions (UTRs).

### *Diplonema* cultures and YFP-tagging of genes at the endogenous locus

All cell lines used in this study were derived from *Diplonema papillatum* (ATCC 50162) and are listed in Table S1. Cells were grown at 27°C in artificial sea water containing 36 g/l Instant Ocean Sea Salt (Instant Ocean), 1 g/l trypton (Formedium, TRP01), and 1% fetal bovine serum (Merck, F9665) in vented flasks. For transfection of C-terminal YFP-tagging constructs, ∼5 to 10 μg plasmids were linearized by *Not*I or other enzymes, cleaned up by ethanol precipitation, resuspended in transfection reagent (Ingenio Electroporation Kit for the EZporator Electroporation System, Cambridge Biosciences), and transfected into 2×10^7^–3×10^7^ cells using Amaxa Nucleofector IIb (Lonza Bioscience) as previously described (Faktorová et al., 2026). Populations of transfected cells were obtained by addition of 75 μg/ml G418 (Merck).

### Microscopy

To observe native YFP signals without detergent extraction, cells fixed in formaldehyde were imaged as previously described with minor modifications (Akiyoshi et al., 2025). Briefly, 1–2 ml cell culture was centrifuged at 2000 ***g*** for 3 min. Cells were fixed by 4% formaldehyde solution (Life Technologies, 28906) diluted in PBS for 5 min, rinsed with PBS twice, resuspended in a small volume (∼10 μl) of DABCO mounting medium [1% (w/v) 1,4-diazabicyclo(2.2.2)octane, 90% glycerol, 50 mM sodium phosphate pH 8.0] with 100 ng/ml DAPI, and mounted onto glass slides. To observe native YFP signals on detergent-treated cells, 2 ml cell culture was centrifuged at 2000 ***g*** for 3 min. The pellet was treated with PEME (100 mM PIPES-NaOH 6.9, 2 mM EGTA, 1 mM MgSO_4_ and 0.1 mM EDTA) with 1% NP-40 and immediately centrifuged at 2000 ***g*** for 3 min, followed by fixation and slide preparation as described above. Images were captured on an Axioimager.Z2 microscope (Zeiss) installed with ZEN using a Hamamatsu ORCA-Flash4.0 camera with 63× objective lenses (1.40 NA). Typically, 15–25 *z*-sections covering 3–6 μm were collected. Images were analyzed in ImageJ/Fiji (Schneider et al., 2012). Figures were made in Inkscape (version 1.4, https://inkscape.org/).

### U-ExM

Ultrastructure expansion microscopy of *D. papillatum* was carried out by adapting protocols used in *T. brucei* (Amodeo et al., 2021; Gorilak et al., 2021). 1 ml cell culture was centrifuged at 2000 ***g*** for 3 min. Cells were fixed in 4% formaldehyde solution diluted in PBS for 5 min, followed by centrifugation. The pellet was resuspended in 500 μl of fixative solution [0.7% formaldehyde, 1% acrylamide (Merck, A4058) in PBS]. Cells were settled onto a 12 mm coverslip (Thermo Fisher Scientific, no. 1.5, CB00120RA120MNZ0) overnight at room temperature. Gelation was performed on a glass slide wrapped with parafilm on a flat metal surface in ice bucket. 50 μl of the monomer solution [19% sodium acrylate (Fluorochem, 461652), 10% acrylamide and 0.1% N, N′-methylene-bis-acrylamide (Bis) (Merck, M7256) in PBS] chilled on ice was quickly mixed with 0.25 μl of TEMED (MP Biomedical, 04805613) and 2.5 μl of 10% ammonium persulfate (Thermo Fisher Scientific, 17874), and put on the parafilm. Immediately after this, the coverslip which cleared excess liquid (with the cells facing down) was placed on the monomer solution and incubated for 5 min. Then the slide was moved to a wet chamber and incubated at 37°C for 30 min to promote gel polymerization.

The parafilm was cut into pieces to move the gel/coverslip to a six-well plate containing 2 ml of denaturation buffer (50 mM Tris-HCl pH 9.0, 200 mM SDS, 200 mM NaCl) to promote detachment of the gel from the coverslip. The gel was then transferred to a 1.5 ml tube containing 1 ml of denaturation buffer, and incubated at 95°C for 60 min. Then the gel was moved into a 12 cm square Petri dish containing 10–20 ml MilliQ water and expanded by three changes into fresh MilliQ water. After checking the expansion factor (typically 4.0–4.2), the gel was cut into smaller pieces (∼1 cm×1 cm square) for immunostaining. The gel piece was moved to a six-well plate and shrunk in PBS for 30 min. Gels were incubated with 600 μl of primary antibodies [rabbit polyclonal anti-GFP antibody that also recognizes YFP (OriGene, TP401, 1 mg/ml, used at 1:500 dilution) and mouse monoclonal anti-α-tubulin antibody (Merck, T5168, used at 1:200 dilution) diluted in PBS with 1% BSA] overnight on a rocking platform at room temperature. Gels were washed with 4 ml PBS for 15 min, three times, followed by incubation with secondary antibodies [goat Alexa Fluor 488 anti-rabbit IgG (H+L) (Thermo Fisher Scientific, A11008, used at 1:200 dilution) and goat Alexa Fluor 568 anti-mouse IgG (H+L) (Thermo Fisher Scientific, A11004, used at 1:200 dilution) diluted in PBS with 1% BSA] overnight in dark on a rocking platform at room temperature. Gels were washed with PBS for 10 min, three times. Gels were expanded with 4 ml MilliQ with 0.2% propyl gallate (Merck, P3130) for 15 min, three times. Gels were mounted onto glass-bottom chambers 35 mm (no. 1.5, Thistle Scientific, IB-81218-200) coated with poly-L-lysine (Merck, P4707). Images were captured on an Axioimager.Z2 microscope (Zeiss) installed with ZEN using a Hamamatsu Flash 4 sCMOS camera with 63× objective lenses (1.40 NA). Typically, 167 *z*-sections covering 25 μm were collected.

### Positive staining and TEM

Carbon Films on 400 mesh gold grids (Agar Scientific, AGS160A4) were glow-discharged for 2 min (PELCO easiGlow, 25 mA current) before being deposited carbon-side down on 20 μl 0.01% poly-L-lysine (Sigma, P4707) droplets and incubated for 20 min at room temperature. Grids were then washed three times by dipping in 30 μl droplets of MilliQ water, the residual water was removed [in this and subsequent steps by dabbing the side of the grids with Whatman Grade 1 filter paper (Cytiva)], and placed carbon-side up on a glass slide. *Diplonema papillatum* cell culture was then added to the slide and incubated at room temperature for 75 min. Cell culture was then removed, and grids were washed three in 30 μl droplets of PEME (100 mM PIPES-NaOH 6.9, 2 mM EGTA, 1 mM MgSO_4_ and 0.1 mM EDTA). Residual PEME was removed before adding 6 μl 1% NP40 (diluted with PEME from 10% NP40 stock solution in MilliQ water) and incubating for 1 min at room temperature. Then, residual liquid was removed before grids were deposited on 30 μl droplets of 2.5% glutaraldehyde (25% stock solution diluted with PEME) and incubated for 20 min at room temperature. Grids were then washed five times in 30 μl PEME droplets and residual PEME removed, before 6 μl 2% uranyl acetate was added to the grids and incubated for 30 s. Then, they were washed five times in 30 μl MilliQ water droplets, residual water removed, and grids deposited onto filter paper in a glass Petri dish and left to dry overnight at room temperature. TEM images were acquired using a 120 kV JEOL TEM-1400 Plus with a Gatan OneView camera using Digital Micrograph version 3.61.4719.0. The images in Fig. 1 were both taken with an exposure time of 1 s. The image in Fig. 1B was taken at a magnification of 5kX with −4.813 μm defocus and the image in Fig. 1C was taken at a magnification of 4kX with −9.626 μm defocus.

### Bioinformatics analysis

Protein sequences were retrieved or accessed from UniProt (UniProt Consortium, 2019), TriTryp (Aslett et al., 2010), EukProt (Richter et al., 2022) or published studies (Valach et al., 2017, 2023; Butenko et al., 2020; Kaur et al., 2020; George et al., 2022). To look for potential homologs, all publicly available transcriptomes were considered (*Diplonema ambulator*, *Diplonema aggregatum*, *Diplonema japonicum*, *Rhynchopus humris*, *Rhynchopus euleeides*, *Lacrimia lanifica*, *Flectonema neradi*, *Sulcionema specki*, *Artemidia motanka*, *Namystynia karyoxenos* and *Hemistasia phaeocysticola*). Homologs were aligned using MAFFT (L-INS-i, version 7) (Katoh et al., 2019) and visualized with the Clustalx coloring scheme in Jalview (version 2.11) (Waterhouse et al., 2009). Homology searches were done using BLAST in TriTryp (Aslett et al., 2010), EukProt v3 BLAST server (Richter et al., 2022), or hmmsearch using manually prepared HMM profiles (HMMER version 3.0) (Eddy, 1998). Domains were searched using HHpred (Zimmermann et al., 2018) or Foldseek (van Kempen et al., 2024) of AlphaFold2 structural models (Jumper et al., 2021; Abramson et al., 2024; Benz et al., 2024). Pairwise alignment analysis was done using EMBOSS Needle (Rice et al., 2000).

BILBO1 homologs in *D. papillatum* were identified as follows. We first searched BILBO1 homologs in diverse kinetoplastids (*Bodo saltans*, *Trypanoplasma borreli*, *Azumiobodo hoyamushi*, *Papus ankaliazontas* and *Apiculatamorpha spiralis*). We then performed multiple sequence alignment together with *T. brucei* BILBO1 using MAFFT (Katoh et al., 2019), from which a BILBO1-NTD hidden Markov model was created (corresponding to 1–123 residues of TbBILBO1) using HMMER (Eddy, 1998). We then performed hmmsearch against *D. papillatum* protein database (Valach et al., 2023; Gray et al., 2025), identifying 76 hits (BILBOD1–BILBOD 76). Ten additional homologs (BILBOD77–BILBOD86) were identified using full-length BILBO1 protein sequences.

## Supplementary Material



10.1242/joces.264933_sup1 Supplementary information

Table S1. List of *D. papillatum* cell lines, plasmids, and primer sequences used in this study
