# Supplementary material for: Identification of feeding apparatus components in a heterotrophic marine flagellate
Source: J Cell Sci. 2026 Sep 18;139(21):jcs264933. doi: 10.1242/jcs.264933 (PMC13615690; doi:10.1242/jcs.264933)
Supplement: Supplementary file 1 [file joces-139-264933-s1.pdf]

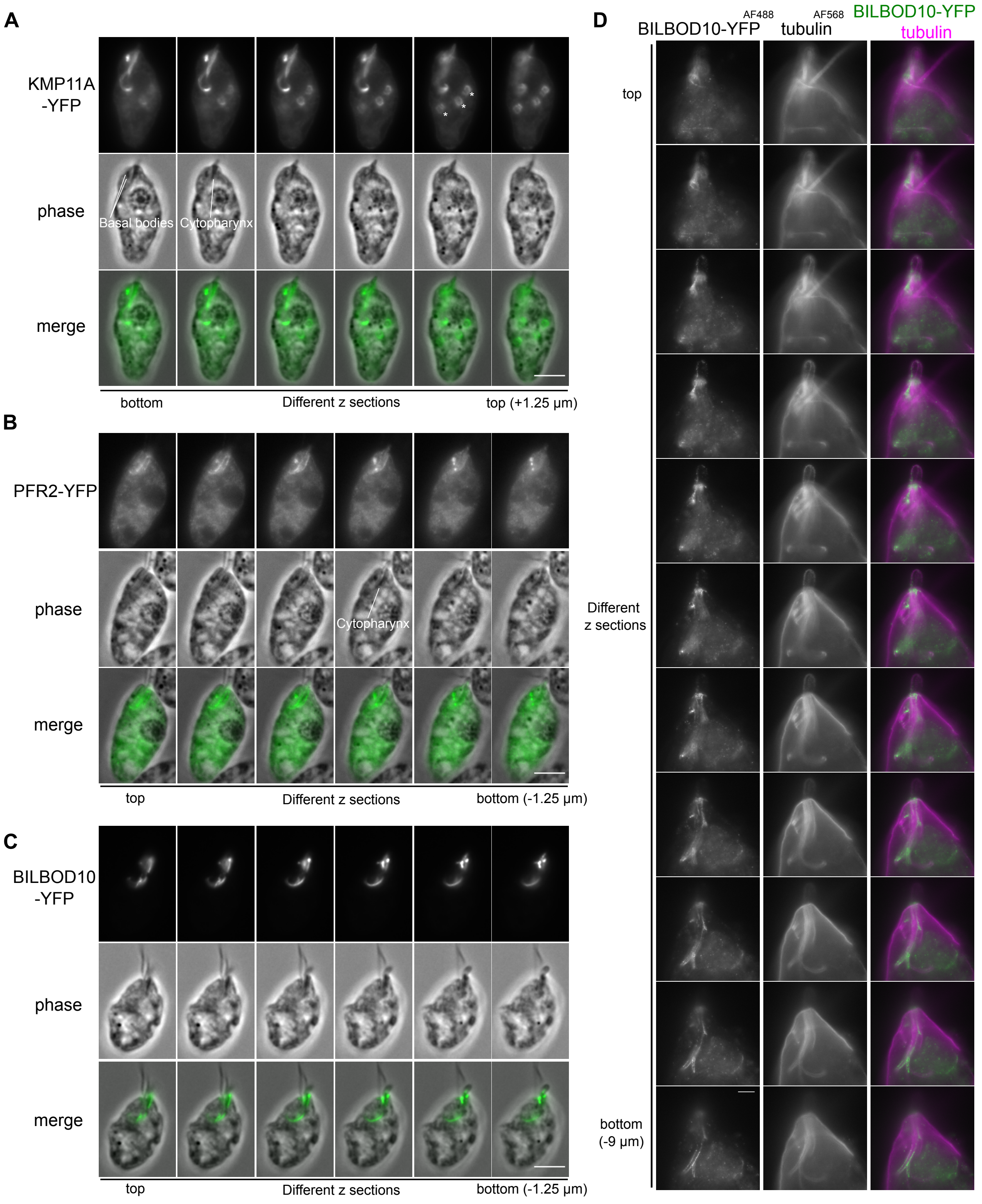

Fig. S1. Additional images of KMP11A, PFR2, and BILBOD10

(A) KMP11A-YFP. (B) PFR2-YFP. (C) BILBOD10-YFP. (D) U-ExM of BILBOD10-YFP. Scale bars, 5 µm.

Journal of Cell Science • Supplementary information

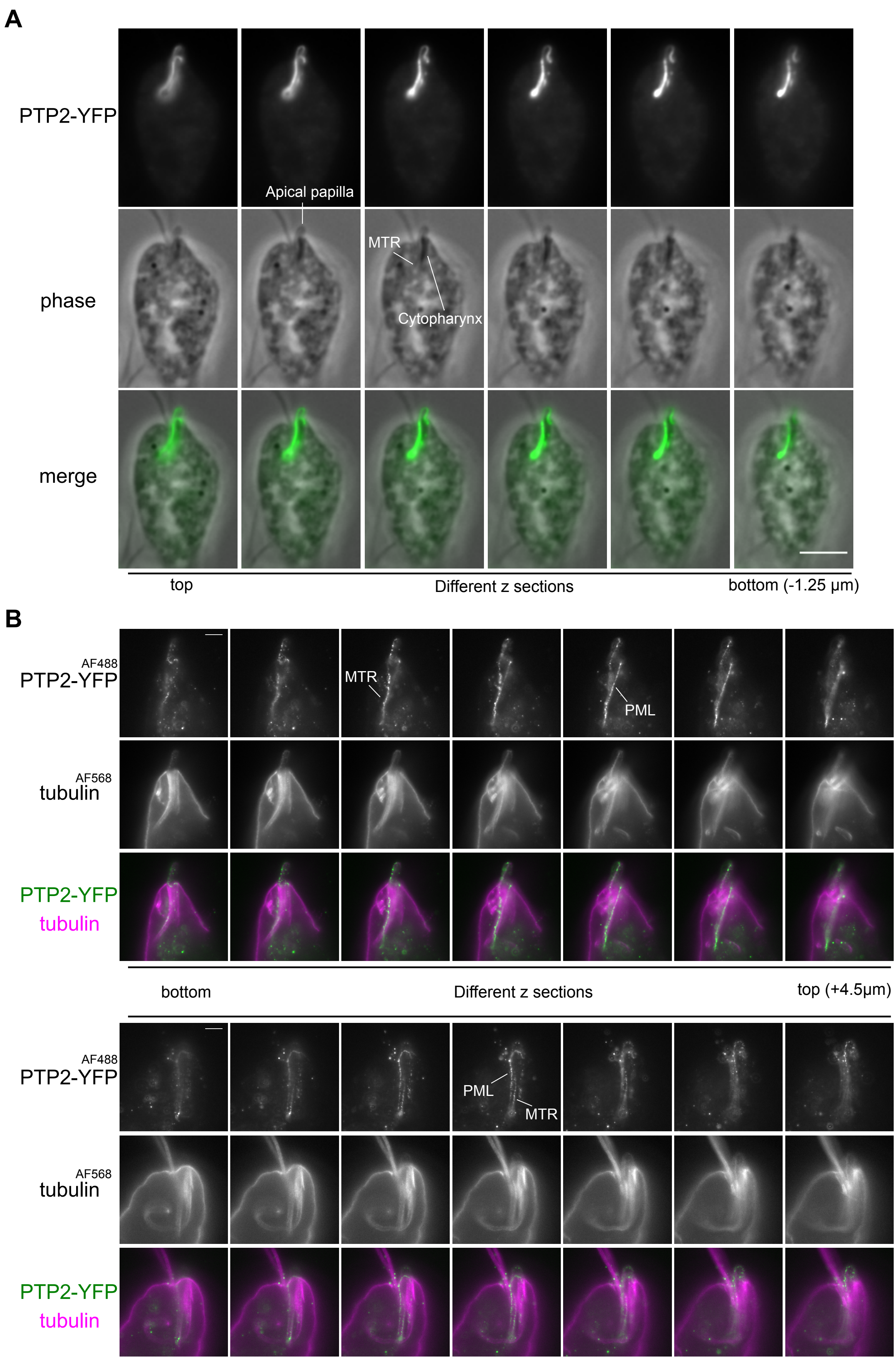

Fig. S2. Additional images of PTP2-YFP.

(A) A cell expressing PTP2-YFP. (B) U-ExM of PTP2-YFP. Scale bars, 5 μm.

Table S1. List of *D. papillatum* cell lines, plasmids, and primer sequences used in this study

Available for download at
https://journals.biologists.com/jcs/article-lookup/doi/10.1242/jcs.264933#supplementary-data

Journal of Cell Science • Supplementary information
