# Supplementary material for: Identification of feeding apparatus components in a heterotrophic marine flagellate
Source: J Cell Sci. 2026 Sep 18;139(21):jcs264933. doi: 10.1242/jcs.264933 (PMC13615690; doi:10.1242/jcs.264933)
Supplement: Supplementary file 2 [file joces-139-264933_review_history.pdf]

Journal of Cell Science

Identification of feeding apparatus components in a heterotrophic marine flagellate

Gillian Clifford, Samuel J.P. Taylor, Midori Ishii, Fernanda Cisneros-Soberanis and Bungo Akiyoshi
DOI: 10.1242/jcs.264933

Editor: Giulia Zanetti

Review timeline

Original submission:	31 March 2026
Editorial decision:	4 May 2026
First revision received:	25 June 2026
Editorial decision:	13 July 2026
Second revision received:	1 August 2026
Accepted:	3 August 2026

Original submission

First decision letter

MS ID#: jcs.264933

MS TITLE: Identification of feeding apparatus components in a heterotrophic marine flagellate

AUTHORS: Gillian Clifford; Samuel J.P. Taylor; Midori Ishii; Fernanda Cisneros-Soberanis; Bungo Akiyoshi

ARTICLE TYPE: Tools and Resources

Dear Dr Akiyoshi,

We have now reached a decision on the above manuscript.

As you will see, the reviewers gave favourable reports but raised some critical points that will require amendments to your manuscript. I hope that you will be able to carry these out because I would like to be able to accept your paper, depending on further comments from reviewers.

Reviewer 1

SUMMARY OF THE ADVANCE MADE IN THIS PAPER AND ITS POTENTIAL SIGNIFICANCE TO THE FIELD

In this manuscript, the authors describe the localisation of 17 proteins to the feeding/flagellar apparatus of the important but understudied organism Diplonema, which are highly abundant and widespread in our oceans. This is an excellent foundational piece of work that will underpin future molecular studies of this and related organisms. The work is of high-quality, using advanced microscopy approaches such as ultra expansion microscopy and clearly described. I have no major concerns but do have a set of minor comments.

SUGGESTIONS TO AUTHORS

Minor comments

© 2026. Published by The Company of Biologists under the terms of the Creative Commons Attribution License (https://creativecommons.org/licenses/by/4.0/).

The first sentence of the introduction does not quite make sense; can you rephrase it.

If the cytostome is on the right hand side of the flagellar pocket, more axes need to be described to orient the reader appropriately.

A schematic of the rootlet system as part of the introduction would be useful to complement the whole mount cytoskeletons. Or alternatively a schematic as a summary figure to show the different structures associated with the feeding/flagellar apparatus and the proteins which have been found to localise to them.

It would be more appropriate to describe detergent lysing the cells not bursting them (p4).

How does the detergent treatment, cause detachment of the feeding apparatus? Are additional salt treatments - NaCl, CaCl2 required? In the TEM the microtubule corset appears intact but in many of the light microscopy images there is just the feeding apparatus. Does this sometimes just happen during preparation, a comment in the text would be useful to help the reader understand the approach.

Do any of the proteins such as Mad2 or POLO1 show any cell cycle dependent change in localisation? POLO2 was not present in all cells, which might be indicative of a cell cycle dependent change but were these cell lines clonal - it is not clear from the methods.

Extending from that the analysis of all proteins has not been considered from a cell cycle angle - have the authors examined how these protein change during the cell cycle and does that provide any insights into how these rootlet structures duplicate and divide? Did the authors confine themselves to one cell cycle phase, if so can that be noted in the text.

Was there any rationale for choosing to tag BILBOD10 and BILBOD26?

The authors should add in that when T. brucei BILBO1 was expressed in an heterologous system rings of tagged BILBO1 were seen and that this could be related to the propensity of this protein to form ring structures.

In Figure 2B, there are clear Mad2-YFP signals but there do not appear to be structures in the phase channel at that position - would changes in the contrast help to show them more clearly.

<u>Reviewer 2</u>

Diplonemids have been turned into genetically tractable organisms only relatively recently, which was a key moment for their more in-depth, i.e. other than morphological and sequencing studies. As stated in the introduction, they are extremely diverse and abundant in the world ocean, and recently emerged as also very informative from the evolutionary point of view. Still, only a few groups committed to their studies because the community still considers them, at least that's what this reviewer thinks, as difficult organisms to work with.

The present study provides not only a lot of novel data but also has the potential to persuade the community that genes can be tagged and sublocalized in a pretty straightforward way, and that these are very interesting protists to study. The quality of the results is very high and the figures are excellent. Some of the findings, in particular the localization of Mad2, MBP65, PFR2, the presence of 86 Bilbo-like proteins, all proteins localized into the feeding apparatus are exciting and will be of interest to the trypanosomatid community.

I have only several minor issues that the authors can easily deal with:

1/ Abstract - heterotrophic unicellular flagellates - a mistake, as these are biflagellars 2/ the end of the abstract sounds awkward. Perhaps something like ....highly abundant marine planktonic organisms"?
3/ I suggest the authors consider the more recent reference to Williamson et al 2025 as more suitable than Burki et al., 2020 (or along with it)

© 2026. Published by The Company of Biologists under the terms of the Creative Commons Attribution License (https://creativecommons.org/licenses/by/4.0/).

4/ page 3 - Cavalier-Smith, 2016 is not an appropriate reference for the diseases caused by trypanosomes, add a more fitting one, there is plenty of them 5/ page 3 - T. brucei and Leishmania spp. is correct 6/ page 4 - I have a question why the method is called ultrastructure expansion microscopy when there is no higher magnification than with standard ExM (perhaps just mny naivite)
7/ when the authors state "highly conserved in diplonemids" - did you check all available genomes? If so, state it somewhere 8/ in the Etheridge reference italicize T. cruzi

Reviewer 3

SUMMARY OF THE ADVANCE MADE IN THIS PAPER AND ITS POTENTIAL SIGNIFICANCE TO THE FIELD

The authors have successfully tagged multiple genes in P. papillatum, a cultured representative of diplonemids, which are currently regarded as one of the most diverse and abundant marine microeukaryotes. Despite their ecological importance, relatively little is known about their cell biology. Although protists (unicellular eukaryotes) account for most of the eukaryote diversity, they often remain understudied. I consider this work to be important for advancing our understanding of eukaryotic biology and evolution. I would also like to emphasize that developing tagging strategies for non-model organisms, as well as establishing protocols for immunostaining and expansion microscopy, is far from trivial. Diplonemids possess complex feeding and and flagellar apparatuses that have been studied in detail by electron microscopy; however, their protein composition has not been previously explored. This study therefore provides an important first step in addressing this gap.

SUGGESTIONS TO AUTHORS

Major comments:

Despite a strong potential of this study, I believe that additional evidence is required to support some of the conclusions. First, any labels identifying the components of the flagellar and feeding apparatuses are missing from most figures. Clear labelling of these structures is essential to demonstrate co-localisation with the tagged proteins. The cell lines analysed using U-ExM and tubulin immunostaining (Mad2, POLO1, BILBOD10, and PTP-2) provide convincing evidence for subcellular localisation, as the relevant structural components can be readily identified. However, for cell lines lacking detergent treatment and additional tubulin staining, the identification of these structures is less clear. For example, it is unclear how the authors identified basal bodies in Figs. 4A and 8A in the absence of tubulin staining and visible flagella in phase-contrast images. Similarly, it is not clear how the structure in Fig 8C was identified as the cytopharynx, or the structure in Fig 8B as the MTR.

To address these issues, I suggest the following:
1) provide tubulin staining for all cells lines, and where necessary, perform additional expansion microscopy
2) clearly label all identifiable components of the flagellar and feeding apparatuses in each panel To avoid overcrowding, structures could be indicated using arrows, arrowheads, asterisks, or letter annotations.
3) include larger images of cells in Figs 5, 6, and 9
4) provide full Z-stacks as Supplementary Videos

Second, the cell cycle stage of the analysed cells is not addressed. In Euglenozoa, the flagellar and feeding apparatuses undergo significant rearrangements during the cell cycle, and protein localisation can vary accordingly. It is therefore important to clarify whether the analyzed cells represent a single stage (e.g. G1) or a mixture of stages.

If possible, the authors should infer the cell cycle stages based on DAPI staining and cytoskeletal markers using tubulin immunostaining (basal body number, flagellum duplication, etc.)
If this is not feasible, the absence of cell cycle information should be explicitly discussed as a limitation.

© 2026. Published by The Company of Biologists under the terms of the Creative Commons Attribution License (https://creativecommons.org/licenses/by/4.0/).

Minor comments

Title: also refer to flagellar apparatus components

All Figures: indicate whether signals correspond to YFP autofluorescence or immunostaining

For each tagged protein: briefly summarise known functions in trypanosomatids and/or other eukaryotes

Abstract: replace "abundant marine plankton" with "abundant marine protists" or "abundant marine microeukaryotes"

Keywords: replace 'eating' with 'feeding' or omit Species name: Paradiplonema papillatum

Additional figure: consider including a schematic diagram of the tagging vector, along with at least one representative construct.

P3 L16: consider replacing "widespread presence" with "ecological importance"
P3 L23: protein composition P4 L3: is the function of Mbp65 known? What is the role of the microtubular quartet in trypanosomatids?
P4 L39: please elaborate on functions of POLO P5 L1: what is known about the function of KMP11?
P5 L10-12: Please, provide a reference for "KMP11A has 87% sequence identities to the T. brucei KMP11 protein, showing an extremely high level of similarity"
P5 L31-54: please, briefly summarise the roles of BILBOD in trypanosomatids P6 L10-12: where do these usually localize in trypanosomatids and/or other eukaryotes?
P6 L19: "feeding and flagellar apparatuses"

Figures and figure legends:

Figure 1:
- magnification details are unnecessary, scale bars suffice
- improve contrast of text labels
- indicate basal bodies and label all visible structures same as for "cytostome" and "PML"
- avoid repeating methodological details already described
- indicate the connection between the MTR tip and cytopharynx (not obvious)

Figure 2:
- did you mean anti-YFP antibody? See also P9 L39
- specify whether signals represent autofluorescence or immunostaining
- label all identifyable structures
- indicate DR, IR, and VR in panel 2D

Figure 3:
- clarify localisation of YFP signal in Cell2
- point to the flagellar roots; the VR is barely discernible, can the image be enhanced?
- there also appears to be weaker signal in the VR and basal bodies; were intensity thresholds applied? If not, how was localisation restricted to the DR?

Figure 4:
4AB: please, include additional Z sections showing flagella for Cell1 to support the identification of basal bodies; can you provide anti-tubulin IFA for the POLO cell line?
4A Cell2 - label all visible components of the flagellar and feeding apparatuses

Figure 5:
Label all structures (basal bodies, flagella, the apical papilla, and MTR) mentioned in the text (P5 L12-16)

Figure 6:
Label all identifiable structures 6B: some scale bars are missing

© 2026. Published by The Company of Biologists under the terms of the Creative Commons Attribution License (https://creativecommons.org/licenses/by/4.0/).

Figure 7:
Label all identifiable structures

Figure 8:
8A: add tubulin immunostaining 8D: comment on signal differences between panels (8D)

Figure 9:
Label all identifiable structures

First revision

Author response to reviewers' comments

We are grateful to the three reviewers for constructive comments on our manuscript. We have addressed their comments below. As requested by the Editor, we also made changes to comply with the journal's format. Briefly, we combined Figure 3 and Figure 4 to reduce the number of figures to 8. We have removed the supplemental material from the Main manuscript file, and also separated the Results and Discussion sections. For the main manuscript file, we provide a "clean" Word file without Tracked changes and a highlighted PDF file with Tracked changes. We made all raw microscopy image files available in FigShare (https://doi.org/10.6084/m9.figshare.32409306).

Reviewer 1:

In this manuscript, the authors describe the localisation of 17 proteins to the feeding/flagellar apparatus of the important but understudied organism Diplonema, which are highly abundant and widespread in our oceans. This is an excellent foundational piece of work that will underpin future molecular studies of this and related organisms. The work is of high-quality, using advanced microscopy approaches such as ultra expansion microscopy and clearly described. I have no major concerns but do have a set of minor comments.

We thank the reviewer for positive comments on our manuscript.

Minor comments

The first sentence of the introduction does not quite make sense; can you rephrase it.

We changed the first sentence from "Obtaining nutrients is a fundamental, universal, and continuous activity for all living organisms with an ecological importance of the planet" to "Obtaining nutrients is a fundamental, universal, and continuous activity for all living organisms".

If the cytostome is on the right hand side of the flagellar pocket, more axes need to be described to orient the reader appropriately.

For the general orientation of the cell, we followed a previous paper (Montegut-Felker and Triemer 1996), which described the fine structure of the feeding apparatus in *Diplonema ambulator* based on electron microscopy analysis. As they noted, however, the exact angle and displacement between the flagellar pocket and the feeding apparatus are not constant due to dramatic cell shape changes (called metaboly). Because of this, we did not include more axes in our manuscript.

A schematic of the rootlet system as part of the introduction would be useful to complement the whole mount cytoskeletons. Or alternatively a schematic as a summary figure to show the different structures associated with the feeding/flagellar apparatus and the proteins which have been found to localise to them.

As suggested, we have added a schematic of the *D. papillatum* cells in Figure 1A.

© 2026. Published by The Company of Biologists under the terms of the Creative Commons Attribution License (https://creativecommons.org/licenses/by/4.0/).

It would be more appropriate to describe detergent lysing the cells not bursting them (p4).

We changed from "to burst the cells" to "to lyse the cells", as suggested.

How does the detergent treatment, cause detachment of the feeding apparatus? Are additional salt treatments - NaCl, CaCl2 required? In the TEM the microtubule corset appears intact but in many of the light microscopy images there is just the feeding apparatus. Does this sometimes just happen during preparation, a comment in the text would be useful to help the reader understand the approach.

For fluorescence microscopy, we performed detergent treatment in solution, fixed, and washed the samples before mounting them on microscopy slides (we did this way because *D. papillatum* cells do not attach to a glass microscope slide). This method efficiently detached the feeding apparatus from the cell body. By contrast, for our TEM analysis, we first attached cells to an EM grid, then performed detergent treatment.
This procedure better maintained the cell architecture, which was important for us to interpret the features of the feeding and flagellar apparatuses. We did not perform any additional salt treatments. Although these details were included in the methods section, we changed the text as follows to give more information in the main text: "We first visualized the feeding apparatus in detergent-extracted *D. papillatum* cells. Cells attached to an EM grid were treated with detergent and imaged by transmission electron microscopy (TEM) (Figure 1B, C)" and "To examine its precise localization, we treated cells with detergent to lyse the cells in a tube before imaging".

Do any of the proteins such as Mad2 or POLO1 show any cell cycle dependent change in localisation? POLO2 was not present in all cells, which might be indicative of a cell cycle dependent change but were these cell lines clonal - it is not clear from the methods.

Mad2 signal is present throughout the cell cycle. POLO1 and POLO2 signals seem cell cycle regulated. However, as the reviewer pointed out, these cell lines are not clonal (because it is laborious to isolate clones for many different proteins). We therefore added the following sentence to the manuscript "It is noteworthy that POLO1 and POLO2 signals were not found in all cells. Although we cannot exclude a possibility of inefficient integration of the YFP-tagging constructs at the correct genomic loci, it is plausible that they are cell cycle regulated. Further work is needed to determine precise locations of POLO2 as well as to reveal their functions and cell cycle regulations".

Extending from that the analysis of all proteins has not been considered from a cell cycle angle - have the authors examined how these protein change during the cell cycle and does that provide any insights into how these rootlet structures duplicate and divide? Did the authors confine themselves to one cell cycle phase, if so can that be noted in the text.

We do not have a good cell cycle marker for *D. papillatum*, making it difficult to infer the cell cycle dependent changes in the localization of individual proteins. Examining the localization changes of these proteins during the cell cycle is therefore beyond the scope of this manuscript.

Was there any rationale for choosing to tag BILBOD10 and BILBOD26?

We tagged these BILBO-like proteins because they were detected by our immunoprecipitation/mass spectrometry of the CLK1 kinase. However, our subsequent experiments showed that these BILBO-like proteins are not specific to the CLK1 immunoprecipitation sample. We did not include these details to avoid confusing readers. As we mentioned in the manuscript, it will be interesting to systematically tag many BILBO-like proteins and examine their localization.

The authors should add in that when T. brucei BILBO1 was expressed in an heterologous system rings of tagged BILBO1 were seen and that this could be related to the propensity of this protein to form ring structures.

We have included the following statement "BILBO1 is a component of the flagellar pocket collar in *T. brucei*, which has polymer-forming properties that allow it to self-assemble into extended

© 2026. Published by The Company of Biologists under the terms of the Creative Commons Attribution License (https://creativecommons.org/licenses/by/4.0/).

filaments" and cited three papers (Bonhivers et al., 2008; Florimond et al., 2015; Zelená et al., 2025).

In Figure 2B, there are clear Mad2-YFP signals but there do not appear to be structures in the phase channel at that position - would changes in the contrast help to show them more clearly.

We changed the contrast but failed to observe clear structures in the phase channel.

Reviewer 2: Diplonemids have been turned into genetically tractable organisms only relatively recently, which was a key moment for their more in-depth, i.e. other than morphological and sequencing studies. As stated in the introduction, they are extremely diverse and abundant in the world ocean, and recently emerged as also very informative from the evolutionary point of view. Still, only a few groups committed to their studies because the community still considers them, at least that's what this reviewer thinks, as difficult organisms to work with.

The present study provides not only a lot of novel data but also has the potential to persuade the community that genes can be tagged and sublocalized in a pretty straightforward way, and that these are very interesting protists to study. The quality of the results is very high and the figures are excellent. Some of the findings, in particular the localization of Mad2, MBP65, PFR2, the presence of 86 Bilbo-like proteins, all proteins localized into the feeding apparatus are exciting and will be of interest to the trypanosomatid community.

We thank the Reviewer for rightly pointing out that many people still think diplonemids as difficult organisms to work with. Driven by our interest in understanding the origin of unique kinetoplastid kinetochores, we started working on *Diplonema papillatum* in 2024 April. It is true that we too had considered it as a difficult organism, and in fact, we encountered many issues even in a simple procedure such as imaging of cells (e.g. they would not adhere to microscopy slides). By streamlining a YFP-tagging protocol, we have managed to tag more than 100 genes during the last two years (mostly by one person), which resulted in the identification of many proteins that localize to the feeding apparatus. Methods to enable conditional protein depletion in *D. papillatum* are under development. In our hands, *D. papillatum* has become as tractable as *Trypanosoma brucei*. It is our hope that more and more people will soon realize that this organism is a highly tractable (and interesting) organism and start working on it. We are happy to share protocols and reagents.

I have only several minor issues that the authors can easily deal with:

1/ Abstract - heterotrophic unicellular flagellates - a mistake, as these are biflagellars

We do not think this is a mistake. We used the term unicellular, not uniflagellar. Regardless, to avoid confusions, we changed to "heterotrophic single-celled flagellates" in the revised manuscript.

2/ the end of the abstract sounds awkward. Perhaps something like ....highly abundant marine planktonic organisms"?

We made the change as suggested.

3/ I suggest the authors consider the more recent reference to Williamson et al 2025 as more suitable than Burki et al., 2020 (or along with it)

The corresponding author of this manuscript, Bungo Akiyoshi, does not think Williamson et al 2025 *Nature* is a more suitable reference because he does not think that phylogenetic analyses using bacterial-origin proteins can determine the root of the eukaryotic tree of life (Akiyoshi, Journal of Cell Science, 2025 and 2026). We therefore used Burki et al. 2020 as a reference.

4/ page 3 - Cavalier-Smith, 2016 is not an appropriate reference for the diseases caused by

© 2026. Published by The Company of Biologists under the terms of the Creative Commons Attribution License (https://creativecommons.org/licenses/by/4.0/).

trypanosomes, add a more fitting one, there is plenty of them

We used Cavalier-Smith 2016 as a reference for diplonemids and their close relationship to trypanosomatids (rather than disease aspects). We have added one review on the disease aspect of kinetoplastids (Field et al. 2017).

5/ page 3 - T. brucei and Leishmania spp. is correct

We made the change as suggested.

6/ page 4 - I have a question why the method is called ultrastructure expansion microscopy when there is no higher magnification than with standard ExM (perhaps just mny naivite)

Ultrastructure ExM (developed by the Hamel/Guichard group) is a modified version of ExM (developed by the Boyden's group). We adapted a ultrastructure ExM protocol, which is thought to be a better method to preserve ultrastructures within cells.

7/ when the authors state "highly conserved in diplonemids" - did you check all available genomes? If so, state it somewhere

Yes, we examined all publicly available transcriptomes of diplonemids (*Diplonema ambulator, Diplonema aggregatum* Dg567, *Diplonema japonicum, Rhynchopus humris, Rhynchopus euleeides, Lacrimia lanifica, Flectonema neradi, Sulcionema specki, Artemidia motanka, Namystynia karyoxenos, and Hemistasia phaeocysticola*) and found a putative homolog of KinesinWW even in *Hemistasia phaeocysticola*, a divergent diplonemid. We included this information in the Methods.

8/ in the Etheridge reference italicize T. cruzi

In the revised manuscript, we italicized organism names including *T. cruzi*.

Reviewer 3:

The authors have successfully tagged multiple genes in P. papillatum, a cultured representative of diplonemids, which are currently regarded as one of the most diverse and abundant marine microeukaryotes. Despite their ecological importance, relatively little is known about their cell biology. Although protists (unicellular eukaryotes) account for most of the eukaryote diversity, they often remain understudied. I consider this work to be important for advancing our understanding of eukaryotic biology and evolution. I would also like to emphasize that developing tagging strategies for non-model organisms, as well as establishing protocols for immunostaining and expansion microscopy, is far from trivial. Diplonemids possess complex feeding and and flagellar apparatuses that have been studied in detail by electron microscopy; however, their protein composition has not been previously explored. This study therefore provides an important first step in addressing this gap.

We thank the referee for the positive assessment on our manuscript and for providing constructive suggestions.

Major comments:

Despite a strong potential of this study, I believe that additional evidence is required to support some of the conclusions. First, any labels identifying the components of the flagellar and feeding apparatuses are missing from most figures. Clear labelling of these structures is essential to demonstrate co-localisation with the tagged proteins. The cell lines analysed using U-ExM and tubulin immunostaining (Mad2, POLO1, BILBOD10, and PTP-2) provide convincing evidence for subcellular localisation, as the relevant structural components can be readily identified. However, for cell lines lacking detergent treatment and additional tubulin staining, the identification of these structures is less clear. For example, it is unclear how the authors identified basal bodies in Figs. 4A and 8A in the absence of tubulin staining and visible flagella in phase- contrast images. Similarly, it is not clear how the structure in Fig 8C was identified as the cytopharynx, or the structure in Fig 8B as the MTR.

© 2026. Published by The Company of Biologists under the terms of the Creative Commons Attribution License (https://creativecommons.org/licenses/by/4.0/).

We found that many cellular features are visible by phase contrast microscopy. For example, we can clearly visualize basal bodies, cytopharynx, and flagella in almost all cells by looking through the z slices of phase contrast images. In addition, MTR is also visible in some cases. We determined the localization of each protein by looking at many cells. When signals remain after detergent treatment, we also used the information to confirm the localization (namely for Mad2, MBP65, KMP11A, BILBOD10, PTP2, KIFC1, KinesinWW, PP1-2, PP1-4, MTR1, PML1, and APL1). For the basal body localization of KIFC1, we added one more image because the original one does not clearly show basal bodies.
We did not find much signal after detergent treatment for POLO1, POLO2, PFR2, BILBOD26, or CENP-E. For POLO1, we managed to determine the localization by expansion microscopy despite the relatively weak signal. For PFR2, the major signal was found to co-localize with basal bodies and cytopharynx which were visible by phase contrast. For BILBOD26, weak signals were found around the feeding apparatus as well as ring-like signals at unknown places. For CENP-E, the signal was found at the end of MTR that was visible in phase contrast images. The localization of POLO2 is highly complicated and we have not been able to obtain images by expansion microscopy due to technical difficulties (low signal and cell cycle regulation). For this reason, we had included the following statement in the original manuscript "Although further work is needed to determine its precise locations".

To address these issues, I suggest the following:

1) provide tubulin staining for all cells lines, and where necessary, perform additional expansion microscopy

As stated above, we can visualize many cellular features (e.g. papilla, basal bodies, flagella, cytopharynx and nucleus in almost all cells as well as MTR in some cells), in our phase contrast images. In contrast, tubulin staining in regular immunofluorescence did not help us to visualize those features already visible by phase contrast. Performing additional expansion microscopy is labour-intensive and is beyond the scope of this manuscript.

2) clearly label all identifiable components of the flagellar and feeding apparatuses in each panel To avoid overcrowding, structures could be indicated using arrows, arrowheads, asterisks, or letter annotations.

We have added labels for relevant structures to explain the localization of indicated proteins. We feel that adding labels for all recognizable features would cause overcrowding and dilute the most relevant messages.

3) include larger images of cells in Figs 5, 6, and 9

We have made images larger in these figures.

4) provide full Z-stacks as Supplementary Videos

We have included all raw TIFF files, rather than Supplementary videos, in FigShare (https://doi.org/10.6084/m9.figshare.32409306). This is due to the size of these files (more than 1 Gb in total) as well as the importance of looking at individual channels.

Second, the cell cycle stage of the analysed cells is not addressed. In Euglenozoa, the flagellar and feeding apparatuses undergo significant rearrangements during the cell cycle, and protein localisation can vary accordingly. It is therefore important to clarify whether the analyzed cells represent a single stage (e.g. G1) or a mixture of stages.

If possible, the authors should infer the cell cycle stages based on DAPI staining and cytoskeletal markers using tubulin immunostaining (basal body number, flagellum duplication, etc.)
If this is not feasible, the absence of cell cycle information should be explicitly discussed as a limitation.

We thank the referee for this important comment. Unlike *Trypanosoma brucei*, we cannot

© 2026. Published by The Company of Biologists under the terms of the Creative Commons Attribution License (https://creativecommons.org/licenses/by/4.0/).

determine the cell cycle stage of cells due to a lack of a good cell cycle marker in *D. papillatum*. In our hands, we cannot confidently use DAPI intensity to distinguish between G1, S or G2 cells. We have included the following statement "Due to the lack of cell cycle markers, we were unable to infer cell cycle stages of individual cells" in the Methods.

Minor comments

Title: also refer to flagellar apparatus components

It is true that we also identified flagellar apparatus components (Mad2, MBP65, POLO1). However, we would like to emphasize the identification of feeding apparatus components in the title of this manuscript. We mention flagellar apparatus components in the abstract.

All Figures: indicate whether signals correspond to YFP autofluorescence or immunostaining

We mentioned in the Methods that the native YFP signals were observed in non-ExM experiments, while the signals in ExM experiments derive from immunostaining. To highlight the immunostaining in ExM experiments, we included AF488 or AF568 labels in revised figures.

For each tagged protein: briefly summarise known functions in trypanosomatids and/or other eukaryotes

We included brief summaries of known functions or localizations for PFR, TbPLK, KMP-11, and BILBO1 in *Trypanosoma brucei*.

Abstract: replace "abundant marine plankton" with "abundant marine protists" or "abundant marine microeukaryotes"

We changed to "abundant marine planktonic organisms", as suggested by another reviewer.

Keywords: replace 'eating' with 'feeding' or omit

We removed the word eating as suggested.

Species name: Paradiplonema papillatum

We used the name *Diplonema papillatum*, which was used in a recent publication from the Lukeš and Burger groups (doi: 10.1186/s12864-025-12233-1), who are the leading figures in the field.

Additional figure: consider including a schematic diagram of the tagging vector, along with at least one representative construct.

We previously described the YFP tagging methods and the vector in detail (Akiyoshi et al. 2025, Faktorová et al. 2026). We therefore do not think it is necessary to include a schematic in this manuscript.

P3 L16: consider replacing "widespread presence" with "ecological importance"

We wanted to emphasize the widespread presence of the cytostome-cytopharynx complex in this sentence. To avoid confusions, we changed as follows: "Despite the widespread presence of the cytostome- cytopharynx complex in flagellates and ciliates, relatively little is known about its protein composition or mechanistic understanding".

P3 L23: protein composition

We made the change as suggested.

© 2026. Published by The Company of Biologists under the terms of the Creative Commons Attribution License (https://creativecommons.org/licenses/by/4.0/).

P4 L3: is the function of Mbp65 known? What is the role of the microtubular quartet in trypanosomatids?

The function of MBP65 remains unknown in *T. brucei*. The microtubule quartet in trypanosomatids is thought to play a critical role in cellular organization, flagellum inheritance, and cell division.

P4 L39: please elaborate on functions of POLO

In *T. brucei*, TbPLK plays various roles including basal body biogenesis and cytokinesis.

P5 L1: what is known about the function of KMP11?

In *T. brucei*, KMP-11 localizes at the flagellum and basal body. Knocking down KMP-11 blocks cytokinesis and kinetoplast segregation. However, its precise molecular function remains unclear.

P5 L10-12: Please, provide a reference for "KMP11A has 87% sequence identities to the T. brucei KMP11 protein, showing an extremely high level of similarity"

This is based on our pairwise alignment analysis of *D. papillatum* KMP11A and *T. brucei* KMP11 using EMBOSS Needle. We have mentioned this in the Methods.

P5 L31-54: please, briefly summarise the roles of BILBOD in trypanosomatids

We had a sentence "BILBO1 is a component of the flagellar pocket collar in *T. brucei*". We now changed it to "BILBO1 is a component of the flagellar pocket collar in *T. brucei*, which has polymer-forming properties that allow it to self-assemble into extended filaments."

P6 L10-12: where do these usually localize in trypanosomatids and/or other eukaryotes?

PTP2 is a homolog of protein tyrosine phosphatases (PTPs), which have diverse functions and localizations depending on specific organisms. In other words, sequence similarities do not necessarily mean their localization or function are conserved in different organisms.

P6 L19: "feeding and flagellar apparatuses"

We made the change as suggested.

Figures and figure legends:

Figure 1:
- magnification details are unnecessary, scale bars suffice

We removed the magnification details as suggested.

- improve contrast of text labels

We have tried out best to improve the contrast of text labels by trying different colors and changing the contrast of images.

- indicate basal bodies and label all visible structures same as for "cytostome" and "PML"

We added labels for basal bodies. Cytostome is not clear in Figure 1B, while PML is not clear in Figure 1A.

- avoid repeating methodological details already described

© 2026. Published by The Company of Biologists under the terms of the Creative Commons Attribution License (https://creativecommons.org/licenses/by/4.0/).

We removed the details.

- indicate the connection between the MTR tip and cytopharynx (not obvious)

We highlighted the connection by asterisks.

Figure 2:
- did you mean anti-YFP antibody? See also P9 L39

We did use an anti-GFP antibody that also recognizes YFP, which is a variant of GFP. We mention this in the Methods.

- specify whether signals represent autofluorescence or immunostaining

As stated above, we mention this in the Methods.

- label all identifyable structures

We have added labels for relevant structures to explain the localization of indicated proteins.

- indicate DR, IR, and VR in panel 2D

We added the labels.

Figure 3:
- clarify localisation of YFP signal in Cell2

We do not yet fully understand the localization of this protein as explained in the text.

- point to the flagellar roots; the VR is barely discernible, can the image be enhanced?

We tried our best to show the VR as clearly as possible. In this case, the DR was visible on the other basal body to confirm that the VR-attached basal body is the ventral basal body.

- there also appears to be weaker signal in the VR and basal bodies; were intensity thresholds applied? If not, how was localisation restricted to the DR?

The signal intensity of POLO1 was reproducibly weak in our U-ExM experiments, likely because of the limited abundance of this protein. In expansion microscopy, the strongest signal was always found on the DR. Although we cannot exclude a possibility that POLO1 also localizes at the VR, the fact that we observed only one major line signal in regular fluorescence microscopy suggests that the primary localization of POLO1 is at the DR.

Figure 4:
4AB: please, include additional Z sections showing flagella for Cell1 to support the identification of basal bodies; can you provide anti-tubulin IFA for the POLO cell line?

We have made the raw TIFF file available in FigShare.

4A Cell2 - label all visible components of the flagellar and feeding apparatuses

We have added labels for relevant structures to explain the localization of indicated proteins.

Figure 5:
Label all structures (basal bodies, flagella, the apical papilla, and MTR) mentioned in the text (P5 L12-16)

© 2026. Published by The Company of Biologists under the terms of the Creative Commons Attribution License (https://creativecommons.org/licenses/by/4.0/).

We have added labels for relevant structures to explain the localization of indicated proteins.

Figure 6:
Label all identifiable structures

We have added labels for relevant structures to explain the localization of indicated proteins.

6B: some scale bars are missing

A missing scale bar is added.

Figure 7:
Label all identifiable structures

We have added labels for relevant structures to explain the localization of indicated proteins.

Figure 8:
8A: add tubulin immunostaining

We did not add tubulin IF as stated above.

8D: comment on signal differences between panels (8D)

We added the following comment "Note that only weak cytopharynx signal is visible in detergent-treated cells" in the legend.

Figure 9:
Label all identifiable structures

We have added labels for relevant structures to explain the localization of indicated proteins.

Second decision letter

MS ID#: jcs.264933R1

MS TITLE: Identification of feeding apparatus components in a heterotrophic marine flagellate

AUTHORS: Gillian Clifford; Samuel J.P. Taylor; Midori Ishii; Fernanda Cisneros-Soberanis; Bungo Akiyoshi

ARTICLE TYPE: Tools and Resources

Dear Dr Akiyoshi,

We have now reached a decision on the above manuscript.

As you will see, the reviewers gave favourable reports but raised some further minor points that will require amendments to your manuscript.

Reviewer 1

SUMMARY OF THE ADVANCE MADE IN THIS PAPER AND ITS POTENTIAL SIGNIFICANCE TO THE FIELD

In this revision the authors have addressed all my concerns and included an additional cartoon and clarified the text. I just have a few minor comments.

© 2026. Published by The Company of Biologists under the terms of the Creative Commons Attribution License (https://creativecommons.org/licenses/by/4.0/).

SUGGESTIONS TO AUTHORS

Minor comments

1) I the summary statment (pg2) include the actual number of proteins (17) as you have in the abstract rather than 'more than ten'

2) In Figure 1A the MTR appears to leave the cell body/membrane, which I think is represented by the thin black line - is this in fact a cartoon of the cytoskeleton? Could the authors clarify or include the apical papilla/MTR within the cell membrane?

3) I think it would be more appropriate to include the caveat about the difficulties of staging the cells within the cell cycle in the results section rather than in the methods.

Reviewer 2

Dear authors,

thank you for significantly improving the manuscript and either accepting the proposed changes or clearly explaining why you chose to leave the original version.

I propose acceptance of the resubmission as it is.

Reviewer 3

SUMMARY OF THE ADVANCE MADE IN THIS PAPER AND ITS POTENTIAL SIGNIFICANCE TO THE FIELD

I thank the authors for carefully addressing the majority of my comments. I believe that the manuscript has been substantially improved. I have only a few remaining minor comments listed below.

SUGGESTIONS TO AUTHORS

Minor comments
P1, L58-61: the components of the feeding apparatus have been described previously in several studies. I believe that this work identifies their protein composition. However, these findings alone do not explain the feeding mechanism. Please consider revising this statement.
P2, L16-19: please refer to proteins, rather than "components"
P5, L23-25: please refer the reader to the sequence alignment (Supplementary File)
P7, L34-35: Discussion, not Discussions P8, L23: Oxford Nanopore sequencing P10, L3: the gel was transferred P10, L21: and incubated overnight on a rocking platform Fig 3A, cell 2: please add labels indicating all visible structures Fig 4A, top panel: I believe # should be referred to as a hash, rather than a sharp.

Second revision

Author response to reviewers' comments

We thank the reviewers for the additional comments. We have addressed them below.

Reviewer 1:

1) I the summary statment (pg2) include the actual number of proteins (17) as you have in the abstract rather than 'more than ten'

We have added the specific number as suggested.

© 2026. Published by The Company of Biologists under the terms of the Creative Commons Attribution License (https://creativecommons.org/licenses/by/4.0/).

2) In Figure 1A the MTR appears to leave the cell body/membrane, which I think is represented by the thin black line - is this in fact a cartoon of the cytoskeleton? Could the authors clarify or include the apical papilla/MTR within the cell membrane?

The thin black line was used to represent the cell body. We have clarified this in the Figure 1 legend. The apical papilla, which is continuous with the MTR, is outside of the cell body.

3) I think it would be more appropriate to include the caveat about the difficulties of staging the cells within the cell cycle in the results section rather than in the methods.

As suggested, we have moved the statement from the methods to the result section that describes the cell cycle regulation of Polo1 and Polo2 (page 5).

Reviewer 3:

P1, L58-61: the components of the feeding apparatus have been described previously in several studies. I believe that this work identifies their protein composition. However, these findings alone do not explain the feeding mechanism. Please consider revising this statement.

Yes, this work has identified “protein” compositions of the feeding apparatus. Our intention was that their identification will allow us to identify even more proteins, which will be key to starting mechanistic characterization of the feeding mechanism.

P2, L16-19: please refer to proteins, rather than "components"

We made the change as suggested.

P5, L23-25: please refer the reader to the sequence alignment (Supplementary File)

We did not include the sequence alignment of KMP11 for brevity. The gene IDs are included in the manuscript so that interested readers can look at the sequences.

P7, L34-35: Discussion, not Discussions

Thanks for noting the typo. We made the change.

P8, L23: Oxford Nanopore sequencing

sWe made the change as suggested.

P10, L3: the gel was transferred

We fixed the typo.

P10, L21: and incubated overnight on a rocking platform

We fixed the typo.

Fig 3A, cell 2: please add labels indicating all visible structures

We added the label for basal bodies as suggested.

Fig 4A, top panel: I believe # should be referred to as a hash, rather than a sharp.

© 2026. Published by The Company of Biologists under the terms of the Creative Commons Attribution License (https://creativecommons.org/licenses/by/4.0/).

We fixed the mistake. Thanks.

Third decision letter

MS ID#: jcs.264933R2

MS Title: Identification of feeding apparatus components in a heterotrophic marine flagellate

Authors: Gillian Clifford; Samuel J.P. Taylor; Midori Ishii; Fernanda Cisneros-Soberanis; Bungo Akiyoshi

Article Type: Tools and Resources

Dear Dr Akiyoshi,

I am happy to tell you that your manuscript has been accepted for publication in Journal of Cell Science, pending standard publication integrity checks.

© 2026. Published by The Company of Biologists under the terms of the Creative Commons Attribution License (https://creativecommons.org/licenses/by/4.0/).
